# Ultrabright NIR‐II Emissive Polymer Dots for Metastatic Ovarian Cancer Detection

**DOI:** 10.1002/advs.202000441

**Published:** 2020-12-23

**Authors:** Xiaobo Zhou, Qiyu Liu, Wei Yuan, Zhenhua Li, Yuliang Xu, Wei Feng, Congjian Xu, Fuyou Li

**Affiliations:** ^1^ Department of Chemistry & State Key Laboratory of Molecular Engineering of Polymers & Institute of Biomedicine Science Fudan University Shanghai 200433 China; ^2^ Department of Obstetrics and Gynecology of Shanghai Medical School & Shanghai Key Laboratory of Female Reproductive Endocrine Related Diseases & Obstetrics and Gynecology Hospital of Fudan University Fudan University Shanghai Shanghai 200011 China

**Keywords:** aggregation‐induced emission, in vivo imaging, NIR‐II fluorescence imaging, ovarian cancer detection, polymer dots

## Abstract

Intraoperative diagnosis of metastatic tumors is of significant importance to the treatment of ovarian cancer. NIR‐II fluorescence imaging holds great promise for facile detection of tumor in situ with high sensitivity and resolution. Herein, a kind of NIR‐II fluorescent polymer dots (NIR‐II Pdots) with high brightness is developed for real‐time detection of metastatic ovarian cancer via NIR‐II fluorescence imaging. The NIR‐II Pdots are constructed via the self‐assembly of NIR‐II emissive aggregation induced emission luminogens (NIR‐II AIEgens) and poly (styrene)‐graft‐poly(ethylene glycol) in water. Such NIR‐II Pdots show very high fluorophore contents of nearly 30% and high quantum yield of 5.4% at emission maximum near 1020 nm. Further modification of the NIR‐II Pdots with targeting peptides yields NIR‐II Pdots‐GnRH, which can afford enhanced affinity of NIR‐II Pdots to ovarian cancer. Upon intravenous injection of the NIR‐II Pdots, whole‐body organs and vessels, peritoneal and lymphatic metastases of ovarian cancer are clearly visualized by NIR‐II fluorescence imaging. Under the guidance of NIR‐II fluorescence imaging, the metastatic foci with the diameter down to ≈2 mm can be facilely eliminated. The results indicate preclinical potential value of the NIR‐II Pdots for metastatic ovarian cancer detection.

## Graphical abstract

An effective strategy to construct NIR‐II fluorescent polymer dots (NIR‐II Pdots) with high brightness, superior photostability, and small size (≈20 nm) is reported. By NIR‐II fluorescence imaging, the NIR‐II Pdots is successfully applied to real‐time whole‐body vessel imaging, organs imaging, and detection of metastatic ovarian tumor in vivo.

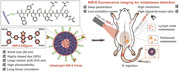

## 1 Introduction

Metastases account for most cancer‐associated deaths around the world. That mainly attributes to the difficulty in early detection and complete surgical resection of metastatic tumors caused by their tiny size and nonspecific histological morphology.^[^ ^1^, ^2^ ^]^ The combination of specific detection of tiny tumor lesions and intraoperative imaging techniques might improve the completeness of surgical resection. Clinical imaging techniques, such as X‐ray, magnetic resonance imaging, computed tomography, ultrasonic imaging, and positron emission tomography are widely used to locate the tumors site preoperatively. However, these approaches are limited in intraoperative diagnosis of tumors due to the limited signal sensitivity, long acquisition time, and unavoidable radiation risks.^[^ ^3^ ^]^ Fluorescence imaging has been considered as a promising approach for intraoperative detection of metastatic tumors due to its advantages including high biosafety and sensitivity, superior resolution, and real‐time observability.^[^ ^4^, ^5^, ^6^, ^7^ ^]^

Recently, second near‐infrared window (NIR‐II, 1000–1700 nm) fluorescence imaging has received much attention in biomedical applications.^[^ ^8^, ^9^, ^10^, ^11^ ^]^ Compared with the conventional fluorescence imaging in the visible and NIR‐I (400–950 nm) regions, NIR‐II imaging exhibits significant advantages of higher spatial resolution and signal‐to‐noise ratio,^[^ ^12^, ^13^, ^14^, ^15^ ^]^ which are the keys of fluorescence imaging for metastases detection in vivo. Up to now, many NIR‐II luminscent materials including single‐walled carbon nanotubes, semiconductor quantum dots (QDs), lanthanide doped nanoparticles, conjugated polymers, organic dyes and organic dye based nanoparticles have been developed as promising candidates for tumor detection.^[^ ^10^ ^]^ However, the application of these NIR‐II luminescent materials for real‐time detection of metastatic tumors in vivo still remains a challenge of low luminescence signal ratio of tumor‐to‐normal tissue (T/N) in tiny metastatic tumor.

In the application of NIR‐II fluorescence imaging for tiny metastatic tumor detection, the T/N ratio is mainly determined by the brightness of a single probe and the accumulation of probes in tumor regions. Unlike solid tumors, tiny metastatic tumors are mostly lack of blood vasculatures and have reduced enhanced permeability and retention (EPR) effect.^[^ ^16^, ^17^ ^]^ So fluorescence imaging probes are difficult to effectively accumulate into metastatic tumor regions. Luminescent materials used for tumor detection with unsuitable sizes are rapidly to be cleared and their accumulation in tumor regions is reduced.^[^ ^18^, ^19^, ^20^, ^21^, ^22^, ^23^, ^24^, ^25^, ^26^ ^]^ In addition, improving the brightness of the reported NIR‐II luminescent probes is difficult due to their limited extinction coefficient (*ɛ*), luminescence quantum yield (*ɸ*), and particle size.^[^ ^27^, ^28^, ^29^ ^]^ Therefore, an excellent NIR‐II probe for intraoperative detection of metastases should be satisfied with suitable size to ensure sufficient accumulation and high brightness of a single probe.

**Scheme 1:**
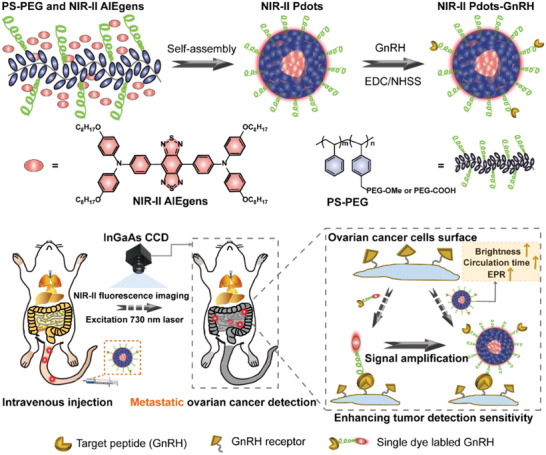
Schematic illustration of the preparation of tumor targeted NIR‐II Pdots and their applications of in vivo metastatic ovarian cancer detection.

Herein, ultrabright NIR‐II emissive polymer dots (NIR‐II Pdots) for real‐time imaging and detection of peritoneal metastases and lymphatic metastases ovarian cancer are developed. The NIR‐II Pdots is a kind of organic nanoparticles (hydrodynamic size ≈20 nm). They are constructed via a self‐assembly process in water due to the hydrophobic interaction between NIR‐II emissive aggregation induced emission luminogens (AIEgens, as luminescent centers)^[^ ^30^, ^31^ ^]^ and poly (styrene)‐graft‐poly(ethylene glycol) (PS‐PEG, as load matrix) (**Scheme** **1**). Unlike most conventional NIR‐II fluorescence dyes with weakened or annihilated fluorescence inside nanoparticles as a result of aggregation‐caused quenching, AIEgens exhibit higher fluorescence quantum yield (QY) in the aggregated state.^[^ ^32^, ^33^, ^34^, ^35^ ^]^ Therefore, the fluorescence QY of the NIR‐II Pdots can remain at a high level of 5.4% when the contents of AIEgens increased to nearly 30%. In addition, the prepared NIR‐II Pdots show higher single probe brightness than other nanoprobes, such as PbS QDs (18 fold),^[^ ^36^ ^]^ NaYF_4_‐5%Nd (4.5 fold), NaYF_4_‐7%Yb 60%Nd (3.3 fold).^[^ ^37^ ^]^ Owing to the high brightness, excellent photostability and long circulation time in blood vasculature of NIR‐II Pdots, real‐time whole‐body imaging in vivo and target imaging of subcutaneous solid tumor with a high T/N signal ratio are realized. Furthermore, an food and drug administration approved peptide sequence for ovarian cancer, tumor‐specific cetrorelix, which can target gonadotropin‐releasing hormone (GnRH) receptors overexpressed on ovarian tumor,^[^ ^38^, ^39^, ^40^ ^]^ is further modified to NIR‐II Pdots. The formed NIR‐II Pdots‐GnRH exhibit enhanced tumor affinity. Upon intravenous injection, both the ovarian cancer peritoneal metastases and lymphatic metastases are clearly visualized by NIR‐II fluorescence imaging. The metastatic foci with diameter down to ≈2 mm can be eliminated under the NIR‐II fluorescence imaging guidance which confirmed by histopathological analysis. Such ultrabright NIR‐II Pdots may be promising to further clinical applications.

## 2 Results and Discussion

### 2.1 Design and Preparation of Ultrabright NIR‐II Pdots

The sensitivity of metastases detection in vivo by fluorescence imaging mainly depends on the T/N of the probe. Therefore, increasing the fluorescence intensity of the metastatic tumor tissue region is advantageous for improving the sensitivity of metastases detection. In order to achieve this goal, both of the luminescence brightness of individual probe and the number of probes accumulated in the metastatic tumor tissue area should be improved. Increasing the number of luminescent centers in a single nanoparticle is the most direct and effective way to increase the luminescence brightness of a single nanoparticle. However, either inorganic nanoparticles or organic nanoparticles, when the size of the nanoparticles is limited, such strategy is no longer applicable because of the aggregation‐caused luminescence quenching. Compared to NIR‐II emissive nanoparticles, NIR‐II emissive organic dyes exhibit lower brightness as a single probe (Table S1, Supporting Information). But, it should be noted that, the sizes of most fluorescent NIR‐II organic dyes are far smaller than the nanoparticles. As a result, NIR‐II emissive organic dyes display higher brightness than the nanoparticles in unit volume. Therefore, the NIR‐II emissive organic dyes with aggregation‐induced emission hold great promise to construct nanoprobes with excellent brightness for NIR‐II fluorescence imaging.

**Figure 1:**
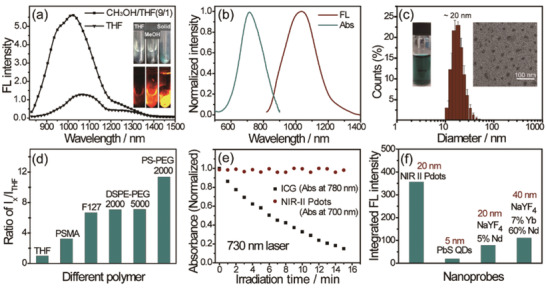
a) Fluorescence spectra of BBTD in THF and THF/methanol mixture (1/9). Inset: NIR‐II fluorescence images of BBTD in THF solution (left), THF/methanol mixture (1/9) (medium), and solid powder (right) under 730 nm illumination (20 mW cm^−2^), respectively. Emission signal collection: 1000 nm long‐pass filter (1000LP), exposure time: 20 ms. b) UV–vis and fluorescence spectra of NIR‐II Pdots in water. c) DLS and TEM images (inset, right) of NIR‐II Pdots, photo of 0.5 mg mL^−1^ NIR‐II Pdots aqueous solutions (inset, left). d) Fluorescence intensity ratio of different amphiphilic polymer loaded NIR‐II AIEgens in water to the NIR‐II AIEgens in THF under an equal concentrations of NIR‐II AIEgens, concentration of NIR‐II AIEgens (BBTD): 2 × 10^−6^ m. e) Photostability of NIR‐II Pdots (0.025 mg mL^−1^) and ICG (20 × 10^−6^ m) in water under continous laser irradiation (730 nm, 50 mW cm^−2^). f) Integrated fluorescence intensity of NIR‐II Pdots, PbS QDs, NaYF_4_‐7%Yb‐60%Nd to NaYF_4_‐5%Nd with equal molar concentrations of nanoparticles in water.

The selected NIR‐II AIEgen triphenylamine‐benzo[1,2‐c:4,5‐c’]bis([1,2,5]thiadiazole) (BBTD) (Scheme 1) shows strong absorption at 760 nm and intense fluorescence at the wavelength beyond 1000 nm in toluene (Figure S1, Supporting Information). In a mixture of tetra‐hydrofuran (THF) and methanol , the BBTD exhibits stronger fluorescence than in THF. In addition, solid state BBTD is significantly brighter than its soluble state in THF (**Figure** **1**a). These results demonstrate the AIE property of BBTD. Compared with traditional NIR‐II dyes like IR1061 which exhibits decreased fluorescence in aggregation state (Figure S2, Supporting Information), such an AIE property of BBTD would be more advantageous to construct nanoprobes with higher concentrations of luminescence centers.

Encapsulating a large number of luminophors in a single nanoparticle via a self‐assembly process between hydrophobic luminophors and amphiphilic polymers is a facile method to construct nanoprobes with high concentrations of luminescence centers. In our design, a kind of amphiphilic polymer (PS‐PEG) with improved capacity to load hydrophobic NIR‐II AIEgens is synthesized (Scheme S1, Supporting Information), and the actual PEG contents in PS‐PEG are determined according to the measurements of ^1^H NMR and GPC characteirzation (Supporting Information). Through nanoprecipitation with PS‐PEG,^[^ ^27^ ^]^ the NIR‐II AIEgens are processed into water‐dispersible dots, termed NIR‐II Pdots. The NIR‐II Pdots show strong absorption maximum near 710 nm with an extinction coefficient of 16.2 L g^−1^, and NIR‐II emission centred at 1020 nm (Figure 1b). The absorption spectra of NIR‐II Pdots in water displays the same absorption shape and maximum absorption peak with BBTD in methanol (Figure S3, Supporting Information), indicating that the BBTD in the Pdots are under the aggregation state. The quantum yield of the NIR‐II Pdots is measured to be 5.4% in water under 730 nm excitation using IR26 as the reference (Figure S4, Supporting Information). The size and morphology of the resulting NIR‐II Pdots are studied using dynamic light scattering (DLS) and transmission electron microscopy (TEM). As shown in Figure 1c, the results demonstrate that NIR‐II Pdots have a spherical morphology with an average diameter of ≈20 nm. Using the same method of preparing the NIR‐II Pdots, different nanoparticles were prepared by replacing the load matrix with different amphiphilic polymers. The optical performances of these nanoparticles are determined (Figure S5, Supporting Information). As shown in Figure 1d; and Table S2 (Supporting Information), the PS‐PEG based NIR‐II Pdots show the smallest size but the highest dye‐to‐polymer mass ratio and fluorescence quantum yield in aqueous solutions. These results indicate that using the synthesized PS‐PEG as the load matrix of the NIR‐II AIEgens is much more appropriate than the other polymers to construct nanoparticles with higher brightness and smaller size. The NIR‐II Pdots in aqueous solution show superior photostability than clinically approved ICG probe under 730 nm laser excitation (Figure 1e; and Figure S6, Supporting Information). The fluorescence and size of the NIR‐II Pdots show ignorable change under different temperatures and medias (Figures S7 and S8, Supporting Information). That indicates the excellent thermal stability of the NIR‐II Pdots. Furthermore, to determine the brightness of the NIR‐II Pdots, the fluorescence spectra with equal mass concentration of NIR‐II Pdots and other NIR‐II emissive nanoprobes (quantum dots and lanthanide nanoparticles, Figure S9, Supporting Information) are measured. The NIR‐II Pdots display brightness of single nanoparticle about 18 times, 3.3 times, and 4.5 times higher than that of PbS QDs, NaYF_4_‐7%Yb60%Nd and NaYF_4_‐5%Nd, respectively (Figure 1f; and Figure S10, Supporting Information).

The NIR‐II Pdots‐GnRH are prepared via the condensation of GnRH peptide and carboxyl‐contained NIR‐II Pdots which are prepared by nanoprecipitation method (Scheme S2, Supporting Information). It should be noted that NIR‐II Pdots‐GnRH and NIR‐Pdots show approximative photophysical properties, size, and morphology (Figures S11 and S12, Supporting Information). The extinction coefficient and fluorescence QY of NIR‐II Pdots‐GnRH are determined to be 15.3 L g^−1^ and 5.5%, respectively. These results indicated that the modification with GnRH peptide does no obvious change to the optical properties and internal structure of NIR‐II Pdots. In addition, the NIR‐II Pdots‐GnRH show approximate diameter and optical properties in different medias, and can keep stable for a long period (Figure S12, Supporting Information). These results confirmed that the prepared NIR‐II Pdots‐GnRH exhibit a high optical stability in different medias.

### 2.2 NIR‐II Pdots for NIR‐II Imaging In Vitro

**Figure 2:**
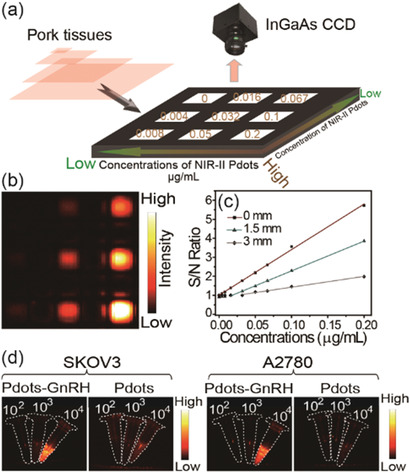
a) Simplified diagram depicting the experimental setup of the in vitro experiment, the average signal‐to‐noise ratio of every well were collected under different concentrations and different thickness of pork tissue. b) NIR‐II fluorescence image of different concentrations (0–0.2 µg mL^−1^) of NIR‐II Pdots without cover of pork tissue. c) Corresponding plots of the S/N ratio value as a function of the concentrations of NIR‐II Pdots under the cover of different thickness (0–3 mm) of pork tissue. d) NIR‐II fluorescent images of NIR‐II Pdots and NIR‐II Pdots‐GnRH labeled ovarian cancer cells (SKOV3 and A2780) at different concentrations. Excitation: 730 nm; collected signal: 1100LP, 200 ms.

To evaluate the fluorescence sensitivity of NIR‐II Pdots, in vitro imaging experiments are performed under different conditions (**Figure** **2**a–c; and Figures S13–S16, Supporting Information) using a InGaAs camera (Princeton Instruments, detection range 800–1700 nm). NIR‐II Pdots show a very high NIR‐II fluorescence sensitivity with detectable signal (1100LP, 200 ms) down to 0.004 µg mL^−1^ under 730 nm excitation (10 mW cm^−2^) (Figure 2b,c). In addition, after covering the well plates with pork tissues of different thickness, the limited detectable concentrations of NIR‐II Pdots have been determined to be 0.032 µg mL^−1^ (1.5 mm, Figure S14, Supporting Information), 0.1 µg mL^−1^ (3 mm, Figure S14, Supporting Information), 0.4 µg mL^−1^ (4.5 mm, Figure S15, Supporting Information), 3.3 µg mL^−1^ (6 mm, Figure S16, Supporting Information), respectively. NIR‐II fluorescence signal (1100LP, exposure time 200 ms) of NIR‐II Pdots (10 µg mL^−1^) displays a high penetration depth up to 7.5 mm pork tissue with 730 nm excitation (Figure S16, Supporting Information). After separately incubating the NIR‐II Pdots and NIR‐II Pdots‐GnRH to ovarian cancer cells (SKOV3 and A2780, Figure S17, Supporting Information), the NIR‐II Pdots‐GnRH labeled cancer cells show nearly 10 folds higher sensitivity with detectable signal down to 10^3^ cells mL^−1^ than that of NIR‐II Pdots labeled cancer cells (Figure 2d; and Figures S18 and S19, Supporting Information). These results indicate NIR‐II Pdots have high brightness and get enhanced tumor cells affinity after modified with GnRH peptide.

### 2.3 NIR‐II Pdots for Wholebody Imaging In Vivo

The toxicities of NIR‐II Pdots and NIR‐II Pdots‐GnRH are determined before using for biomedical applications. The cytotoxicities of NIR‐II Pdots and NIR‐II Pdots‐GnRH are examined with a standard MTT (methyl thiazolyl tetrazolium) assay. The results demonstrate that both of the NIR‐II Pdots and NIR‐II Pdots‐GnRH display minimized cytotoxicity to SKOV3 cells following 24 h incubation (Figure S20, Supporting Information). In vivo toxicities of NIR‐II Pdots and NIR‐II Pdots‐GnRH are examined at 1, 7, and 28 days postinjection. Pathology analysis via hematoxylin and eosin (H&E) staining prove no morphological changes in all of the tested organs (Figures S21 and S22, Supporting Information). Over this testing period, no obvious differences in body weight or blood markers between the test groups (intravenously injected with NIR‐II Pdots or NIR‐II Pdots‐GnRH) and a control group (intravenously injected with phosphate buffer saline, PBS) can be observed (Figure S23 and Table S2, Supporting Information). These results indicate that the NIR‐II Pdots and NIR‐II Pdots‐GnRH exhibit high biocompatibility for biomedical applications.

**Figure 3:**
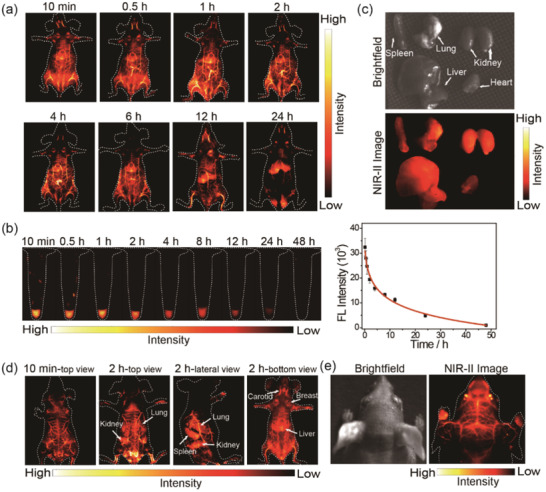
a) Time course of NIR‐II fluorescence images of living mice injected with 200 µL (1 mg mL^−1^) NIR‐II Pdots. Images were acquired at 10 min, 0.5, 1, 2, 4, 6, 12, and 24 h pi, respectively. b) Fluorescence images of blood samples at different time point and the corresponding time course of fluorescence intensity during in 48 h. c) Biodistribution of main organs of NIR‐II Pdots‐treated mice after 48 h postinjection. d) In vivo fluorescence imaging of whole‐body at different imaging view. e) NIR‐II fluorescence images of brain vessels in head (right) and the corresponding brightfield images (left).

Whole‐body vessel imaging in vivo is performed by exciting the intravenously injected NIR‐II Pdots with a 730 nm laser and collecting the NIR‐II fluorescence signal (1100LP, exposure time 200 ms). 10 min after tail vein injection of NIR‐II Pdots solution, the whole‐body vessels could be clearly observed by fluorescence imaging. Importantly, even 6 h after injection of these nanoprobes, the vessels of mouse could still be clearly identified (**Figure** **3**a). In addition, time‐dependent NIR‐II fluorescence intensity of blood is determined to further investigate the biodistribution of NIR‐II Pdots in vivo (Supporting Information). As shown in Figure 3b, the fluorescence intensity of NIR‐II Pdots in blood is decreased with the increasing time. 8 h after intravenous injection, the fluorescence intensity of NIR‐II Pdots in the blood remains at one‐half of the fluorescence intensity of blood that collected at 10 min. Fluorescence imaging in vivo at 24 h (Figure 3a) and the images of organs (Figure 3c; and Figure S24, Supporting Information) show that NIR‐II Pdots are mainly accumulated in livers. The NIR‐II Pdots‐GnRH exhibit similar metabolic kinetics and biodistribution with the NIR‐II Pdots in vivo (Figures S25–S28, Supporting Information). Taking the advantages of the long blood circulation and high brightness of NIR‐II Pdots, different organs located in deep tissues including liver, spleen, lung, and kidneys are clearly observed by fluorescence imaging (Figure 3d). And the brain vessels images are also obtained with high spatial resolution (≈150 µm) (Figure 3e).

### 2.4 NIR‐II Pdots for Subcutaneous Tumor Imaging In Vivo

**Figure 4:**
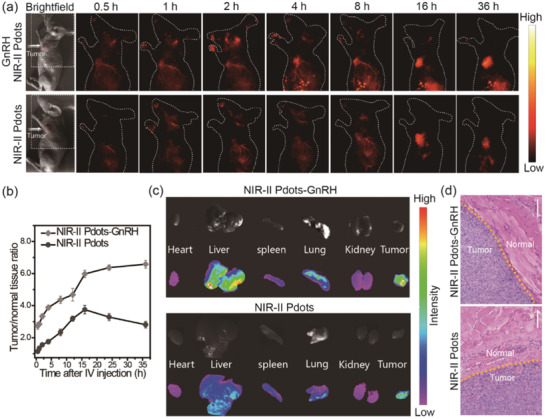
a) Time course of NIR‐II fluorescence images of living tumor‐bearing mice injected with 200 µL (1 mg mL^−1^) NIR‐II Pdots and NIR‐II Pdots‐GnRH. Images were acquired at 0.5, 1, 2, 4, 8, 16, and 36 h pi, respectively. b) Time course of fluorescence intensity during in 36 h. c) Biodistribution of main organs and tumor tissues of NIR‐II Pdots‐treated mice after 36 h postinjection. d) H&E staining of tumor tissue were shown in c), bar: 100 µm.

As the superior performance of the synthesized NIR‐II emissive Pdots for the applications of in vivo imaging, the application of NIR‐II Pdots to subcutaneous tumor imaging are extended. The in vivo experiments are carried out in a murine model with subcutaneous human ovarian adenocarcinoma which constructed by subcutaneous inoculation of human ovarian cancer cells A2780 to mice. Once the tumor size reaches a diameter of ≈0.5 cm, equal amount of NIR‐II Pdots and NIR‐II Pdots‐GnRH (4 mg kg^−1^) are intravenous injected into different mice, respectively. As shown in **Figure** **4**a,b, both NIR‐II Pdots and NIR‐Pdots‐GnRH are tended to accumulate in tumor regions as the time elapsed, which suggest the EPR effect is contribute to the tumor targeting process of these two nanoprobes. Improtantly, according to the time‐dependent T/N ratio of fluorescence images (Figure 4b), NIR‐II Pdots‐GnRH displays more rapid accumulation and longer retention time in tumor tissue region than NIR‐II Pdots. And the T/N ratio of NIR‐II Pdots‐GnRH has been reached ≈6.7, which is much higher than that of NIR‐II Pdots (≈3.9). After dissection of the tumor bearing mice, the tumor tissues show higher fluorescence signals than that of most organs (Figure 4c; and Figures S29 and S30, Supporting Information). The tumor tissues are further confirmed by H&E staining (Figure 4d). These results indicated that the modification of GnRH peptide is facilitate to enhance the affinity of NIR‐II Pdots to ovarian tumors.

### 2.5 NIR‐II Pdots for Metastatic Tumor Imaging In Vivo

**Figure 5:**
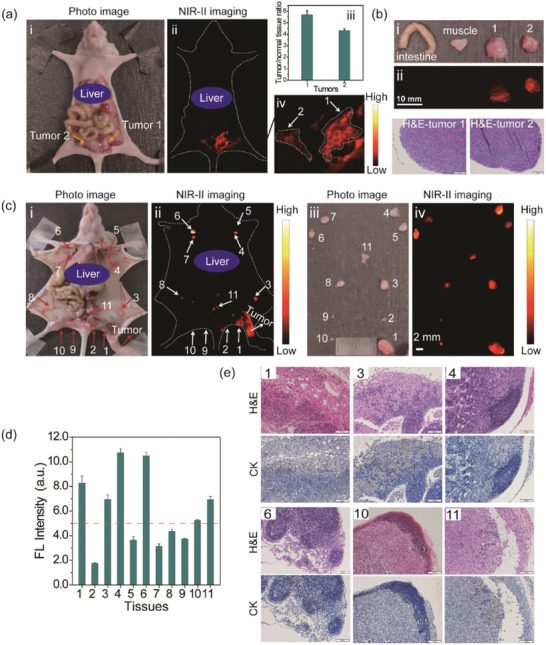
a) Optical photo of human ovarian adenocarcinoma peritoneal metastases model i), the corresponding NIR‐II fluorescence bioimaging results obtained at 48 h PI ii), the enlargement of the NIR‐II nanoprobes labeled large peritoneal metastatic tumors (No. 1 and 2), iii) and the average S/N ratio of peritoneal metastatic tumors iv). b) Optical photos i) and NIR‐II fluorescence images ii) of the resected tumor tissues (No. 1 and 2 shown in (a) from NIR‐II fluorescence imaging guided tumor surgery and normal tissues (muscle and intestine), and the H&E images of tumor tissues. c) Optical photos i) and NIR‐II fluorescence images ii) of ovarian lymphatic metastases mode obtained at 48 h PI and the optical photos iii) and NIR‐II fluorescence images iv) of the resected lymph node tissues (No. 1–11) in vitro. d) Average fluorescence intensity of the resected lymph node tissues shown in (c). e) H&E/IHC staining of lymph node tissue (No. 1, 3, 4, 6, 10, 11 shown in (c) sections. Excitation: 730 nm, Collected signal: 1100 LP.

Encouraged by the tumor imaging results of NIR‐II Pdots‐GnRH on subcutaneous ovarian tumor model mice, peritoneal, and lymphatic metastatic tumor models are further conducted for in vivo stud. (**Figure** **5**). A2780 and ES‐2 tumor bearing mice are used for peritoneal and lymphatic metastatic tumor models, respectively.

The prepared NIR‐II emissive nanoprobes NIR‐II Pdots‐GnRH are intravenous injected into peritoneal metastases tumor bearing mice at the dosage of 4 mg kg^−1^. Then, the NIR‐II fluorescence imaging is performed after injection of nanoprobes for 48 h. As shown in Figure 5a, the tumor bundary is clearly identified by NIR‐II fluorescence imaging and the T/N value of the mestastic tumor regions is as highly as 5.5. After the resection of the metastatic tumors under the guidance of NIR‐II fluorescence imaging (Figure 5b; Figures S31 and S32, Supporting Information), H&E and immunohistochemistry (IHC) staining analysis are performed. The results demonstrate the precise resection of tumors and the experssion of GnRH receptor (GnRHR) in ovarian cancer tissue. Therefore, tiny size (≈2 mm) metastatic ovarian tumor lesions could be clearly identified and thoroughly removed using the prepared NIR‐II Pdots‐GnRH. In comparison, using the targeting group free NIR‐II Pdots as a probe for NIR‐II fluorescence imaging is unable to identify the tiny peritoneal metastases tumor (Figures S33 and S34, Supporting Information). These results further indicate the accumulation of targeting group free NIR‐II Pdots in tumor regions are mainly attributed to the EPR effect. Therefore, such a nanoprobe with modification of GnRH peptide is advantageous in the application of tiny peritoneal metastases tumor detection.

Unexpectedly, after intravenous injection of NIR‐II Pdots‐GnRH to the lymphatic metastases tumor bearing mice, wholebody lymph nodes of the mice are lighted up by NIR‐II fluorescence imaging (Figure 5c). In comparison, after footpad administration of NIR‐II Pdots‐GnRH, only partial lymph nodes of mice could be visualized (Figure S35, Supporting Information). Similar results are also obtained using NIR‐II Pdots as the imaging nanoprobe (Figures S36 and S37, Supporting Information). These results suggest that both the NIR‐II Pdots‐GnRH and NIR‐II Pdots are capable to be ingested into lymph nodes, which might attribute to the nanostructure of Pdots.^[^ ^41^, ^42^, ^43^, ^44^ ^]^ Importantly, under the guidance of NIR‐II fluorescence imaging, the lymph nodes with strong fluorescence signal are selectively resected. All of the lymphatic metastases (NO. 1, 3, and 11) exhibit higher average fluorescence intensity (FL intensity > 5.0) than normal lymph nodes when using the NIR‐II Pdots‐GnRH as the probe via intravenous injection (Figure 5d,e; and Figure S38, Supporting Information). While using the NIR‐II Pdots as the lymphatic metastasis tumor detection probe via intravenous administration or using the NIR‐II Pdots‐GnRH as a probe via footpad administration mode, the fluorescence intensity of normal and tumor metastatic lymph nodes from the same mice have no significant difference (Figures S39 and S40, Supporting Information). In consideration of the indifference accumulation of nontargeting NIR‐II Pdots to lymph nodes, the modification of GnRH peptide may be advantageous to NIR‐II Pdots for selective accumulation in lymphatic metastases. In addition, for the application of lymphatic metastases detection by using NIR‐II Pdots‐GnRH, intravenous administration is superior than local administration, which may due to the low selectivity to matastasis lesions via footpad administration. According to these results, using the NIR‐II Pdots‐GnRH and NIR‐II fluorescence imaing technology, not only the primary tumors in vivo could be observed, but also the microscopic tumor lesions of lymphatic metastases could be detected. More improtantly, such a method could be applied to detect retroperitoneal lymphatic metastases (Figure 5c–e, lymph node No. 11). Such properties of NIR‐II Pdots‐GnRH may help for intraoperative diagnosis of retroperitoneal lymphatic metastases which are common in ovarian cancer patients.

## 3 Conclusion

In summary, ultrabright NIR‐II emissive polymer dots are construted by NIR‐II emissive AIEgens and amphiphilic polymer PS‐PEG for ovarian cancer matastasis detection in vivo. Advantageous from the high loading concentration of AIEgens using PS‐PEG as load matrix, the NIR‐II Pdots exhibit significant enhancement of single particle brightness compared with traditional NIR‐II emissive nanoprobes like QDs and lanthanide luminescence materials. Long blood circulation time of NIR‐II Pdots is obtained due to the hydrophilic PEG coating and small size of NIR‐II Pdots. Further modification with ovarian cancer targeting GnRH peptide, NIR‐II Pdots‐GnRH with enhanced ovarian cancer affinity are obtained. Benefiting from their high brightness and ehanced targeting as well as NIR‐II emission property, upon intravenous injection, the NIR‐II Pdots‐GnRH display superior detection sensitivity during the imaging of ovarian cancer peritoneal metastases and lymphatic metastases. Meanwhile, such a kind of nanoprobes also exhibit additional salient features including high photostability and chemical stability, long tumor retention time and low toxicity. These advantages enable this kind of ultrabright NIR‐II emissive nanoprobes to have great promise for future clinical tumor staging, preoperative diagnosis, and intraoperative navigation.

## 4 Experimental Section

##### Instruments and Methods

The ^1^H and ^13^C NMR spectra were measured at room temperature by using a Bruker Ultra Shield Plus 400 MHz NMR instrument. Matrix assisted laser desorption ionization‐time of flight mass spectra (MALDI‐TOF/TOF) were measured on an AB SCIEX 5800 system. UV–vis absorption spectra were recorded on Lambda 750 (PerkinElmer, America). Photoluminescent spectra were measured using a QM40 (PTI, America) system with a xenon lamp and 730 nm laser as the excitation source. TEM was conducted on a JEOL transmission electron microscope (JEM‐2100) at an acceleration voltage of 100 kV. The number‐average molecular weight (*M*_n_) and weight‐average molecular weight (*M*_w_) of the polymers were characterized in THF by gel permeation chromatography at 35 °C (polystyrene as standard). DLS measurements were taken using a Malvern nanoparticle sizezeta potential analyzer. The NIR luminescence images were collected with an InGaAs‐based NIR camera under an external 730 nm laser.

##### Materials

All chemicals were purchased from commercial sources and used without further purification. All solvents were purified before use. PS–PEG–COOH was purchased from Polymer Source Inc. Ttriphenylamine‐benzo[1,2‐c:4,5‐c’]bis([1,2,5]thiadiazole) (BBTD) was synthesized according to the previous report.

##### Preparation of NIR‐II Pdots

A mixture of BBTD (1 mg), PS‐PEG (2 mg), and THF (2 mL) was sonicated to obtain a clear solution. The mixture was quickly injected into deionized water (20 mL). The mixture was stirred in fume hood for 2 h and concentrated under vacuum. Then, the solution was filtered through a membrane filter (diameter = 0.22 µm) to obtain the NIR‐II Pdots. The NIR‐II Pdots were stored in 4 °C for further usage.

##### Preparation of NIR‐II Pdots‐GnRH

A mixture of BBTD (1 mg), PS‐PEG (1.6 mg), PS‐PEG‐COOH (0.4 mg), and THF (2 mL) was sonicated to obtain a clear solution. The mixture was quickly injected into deionized water (20 mL). The mixture was stirred in fume hood for 2 h and concentrated under vacuum. Then, the solution was filtered through a membrane filter (diameter = 0.22 µm) to obtain the carboxyl contained NIR‐II Pdots. The carboxyl contained NIR‐II Pdots (2 mg) were dispersed in 4 mL of borate buffer (pH = 8.4), followed by dissolution of 1 mg of 1‐(3‐dimethylaminopropyl)‐3‐ethylcarbodiimide hydrochloride. The suspension was stirred for 15 min. Then, 0.2 mg of N‐hydroxysulfosuccinimide sodium salt and 1 mg of GnRH peptide were added. The reaction system was stirred overnight. Finally, the obtained solutions were dialyzed against deionized water for 3 days and concentrated under vacuum. The NIR‐II Pdots‐GnRH were stored in 4 °C for further usage.

##### Fluorescence Stability of Pdots

PL spectra were used to monitor the fluorescence stability of NIR‐II Pdots under continuous 730 nm laser irradiation (50 mW cm^−2^).

##### Stability Test of Nanoprobe in Different Media

NIR‐II Pdots and NIR‐II Pdots‐GnRH were added to ultrapure water, PBS, 10% fetal bovine serum contained PBS (FBS), simulated body fluid (SBF), and 10% FBS contained RMPI 1640 solution to give a final concentration of 0.02 mg mL^−1^, respectively. Then, DLS analysis was performed at different time points, and absorption and emission spectra were also measured at the same time.

##### Cell Culture

Human serous ovarian cancer cell line A2780 and SKOV3, human ovarian clear cell carcinoma cell line ES‐2 were provided by the Institute of Biochemistry and Cell Biology, SIBS, CAS (China). The A2780 cells were grown in RMPI 1640, ES‐2 and SKOV3 were grown in McCOY5A supplemented with 10% FBS at 37 °C and 5% CO_2_. All cells were planted on 14 mm glass coverslips and keep to adhere for 24 h.

##### Tumor Xenografts

Animal experiments were conducted according to the guidelines of the Institutional Animal Care and Use Committee. Tumor cells were harvested when they reached near confluence by incubation with 0.05% trypsin‐EDTA. Cells were pelleted by centrifugation and resuspended in sterile PBS. For subcutaneous tumor model, the SKOV3 cells (5 × 10^6^ cells per site) were implanted subcutaneously into the 4‐week‐old female athymic nude mice. When the tumors reached 0.5 cm in diameter (3 weeks after implantation), the tumor‐bearing mice were subjected to imaging studies. For peritoneal metastases tumor model, the A2780 cells (1 × 10^7^ cells per site) were implanted intraperitoneally into the 4‐week‐old female athymic nude mice. After 2–3 weeks, the tumor‐bearing mice were subjected to imaging studies. For lymphatic metastases tumor model, the ES‐2 cells (1 × 10^6^ cells per site) were implanted subcutaneously into the footpad of the 4‐week‐old female athymic nude mice. After 3–‐4 weeks, the tumor‐bearing mice were subjected to imaging studies.

##### Fluorescence Imaging In Vitro and In Vivo

The luminescent signals from NIR‐II Pdots were collected using InGaAs camera. The system was equipped with a 1100 long‐pass filter (1100LP) for NIR‐II fluorescence imaging of NIR‐II Pdots and NIR‐II Pdots‐GnRH. The excitation source for in vitro and in vivo experiment was an 730 nm laser. At time points of 0.17, 0.5, 1, 2, 4, 8, 12, 24, and 48 h, 50 µL blood was collected using a capillary tube stuck into the corner of one eye, and then diluted with EDTA‐K2 solution. The average signal of solutions and tissues was analyzed by the Bruker imaging software and Origin 8.0 software.

##### In Vitro Biodistribution Analysis

Normal mice were intravenously injected with NIR‐II Pdots or NIR‐II Pdots‐GnRH and euthanized at 1, 6, 12, 24, or 48 h postinjection (*n* = 3). Blood and major organs (heart, liver, spleen, lung, kidney, stomach, intestines, blandder) were collected and then used to NIR‐II fluorescence imaging in vitro. According to the fluorescence intensity of organ tissues and blood samples, the biodistribution of NIR‐II probes at different time were determined.

##### Histological and Hematology Analysis

All animal procedures were in agreement with the guidelines of the Institutional Animal Care and Use Committee, Department of Pharmacy, Fudan University. In the test group, nude mice (*n* = 3) were intravenously injected with NIR‐Pdots and NIR‐Pdots‐GnRH at a total dose of 1 mg mL^−1^ (200 µL). And nude mice (*n* = 3) with no injection were selected as the control group. Tissues and blood samples were harvested from test and control groups after 24 h, 1 and 4 weeks intravenous injection. The collected blood samples were used for hematology and biochemical analysis. The heart, liver, spleen, lung, and kidney were removed, and fixed in paraformaldehyde, embedded in paraffin, sectioned, and stained with hematoxylin and eosin. The sections were observed under an optical microscope.

In fluorescence imaging guided tumor surgery, muscle, intestine, and tumor tissues were harvest and fixed in 4% paraformaldehyde. For IHC analysis, formalin‐fixed, deparaffinised tissues were sectioned into 5 × 10^−6^ m slides. Antigen retrieval was performed using sodium citrate buffer (pH 6) at 95 °C for 20 min. The sections were incubated with primary antibodis: GnRHR (Abcam, ab183079, 1:100), Muc‐1 (Abcam, ab109185, 1:250), CK7 (Abcam, ab181598, 1:8000) overnight at 4 °C, then washing and incubating with secondary antibody for 1 h at room temperature. 3,3’‐diaminobenzidine was used as the chromogen. Finally, the slides were dehydrated and mounted. H&E staining was performed according to a standard protocol. The images were obtained using an optical microscope.

## Conflict of Interest

The authors declare no conflict of interest.

## Supporting information

Supporting Information
