# Supplementary material for: Ultrabright NIR‐II Emissive Polymer Dots for Metastatic Ovarian Cancer Detection
Source: Adv Sci (Weinh). 2020 Dec 23;8(4):2000441. doi: 10.1002/advs.202000441 (PMC7887585; doi:10.1002/advs.202000441)
Supplement: Supplementary file 1 — Supporting Information [file ADVS-8-2000441-s001.pdf]

WILEY-VCH

© 2020 Wiley-VCH GmbH

ADVANCED SCIENCE
Open Access

Supporting Information

for *Adv. Sci.,* DOI: 10.1002/advs.202000441

Ultrabright NIR-II Emissive Polymer Dots for Metastatic Ovarian Cancer Detection

Xiaobo Zhou, Qiyu Liu, Wei Yuan, Zhenhua Li, Yuliang Xu, Wei Feng, Congjian Xu, Fuyou Li**

Supporting Information

Ultrabright NIR-II Emissive Polymer Dots for Metastatic Ovarian Cancer Detection

Xiaobo Zhou, Qiyu Liu, Wei Yuan, Zhenhua Li, Yuliang Xu, Wei Feng, Congjian Xu, Fuyou Li**

Dr. X. B. Zhou, Dr. W. Yuan, Dr. Z. H. Li, Dr. Y. L. Xu, Prof. W. Feng, Prof. F. Y. Li,
Department of Chemistry & State Key Laboratory of Molecular Engineering of Polymers & Institute of Biomedicine Science, Fudan University, Shanghai 200433, China
E-mail: fyli@fudan.edu.cn
Dr. Q. Y. Liu, Prof. C. J. Xu
Department of Obstetrics and Gynecology of Shanghai Medical School & Shanghai Key Laboratory of Female Reproductive Endocrine Related Diseases & Obstetrics and Gynecology Hospital of Fudan University, Fudan University Shanghai 200011, China
E-mail: xucongjian@fudan.edu.cn

Synthesis of PS-PEG

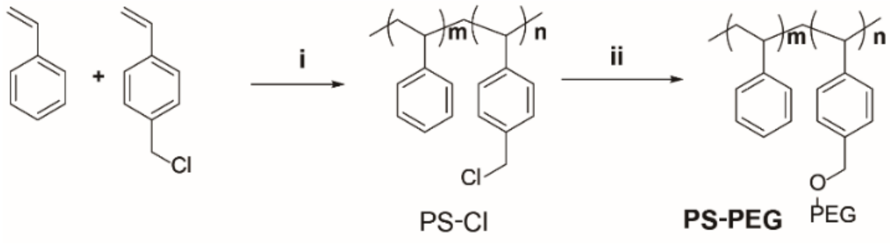

i) AIBN, CTAB, 85 °C, dioxane; ii) NaH, PEG-OH, dry THF, rt.

Scheme S1. Synthetic route of PS-PEG.

PS-Cl: PS-Cl was synthesized using RAFT of styrene and 4-(chloromethyl)styrene. Typically, styrene (2.00 g, 19.2 mmol), 4-(chloromethyl) styrene (0.36 g, 2.4 mmol), chain transfer agent 4-cyano-4-(phenylcarbonothioylthio) pentanoic acid (0.056 g, 0.20 mmol), and initiator AIBN (0.008 g, 0.04 mmol) were mixed in a 25 mL Schlenk flask, and the solution was degassed by three freeze-pump-thaw cycles. The mixture was then heated under argon at 85 °C for 24 h. The polymerization was stopped by cooling the reaction flask in liquid nitrogen. The resulting copolymer was isolated as a pink powder (1.1 g) after dissolving in DCM and precipitating into 40-fold MeOH for three times. ^{1}H NMR (400 MHz, $CDCl_3$, δ) 7.10 (m, 12.26 H), 6.55 (m, 7.38H), 4.55 (bs, 1H), 1.41-2.15 (m, 12.43H), content of Ph-CH_2Cl (10%). GPC (THF, polystyrene standard): M_w 6100, M_n 5700, PDI 1.07.

PS-PEG: PS-Cl (0.1 g), mPEG-OH (Mn~2000) (0.4 g, 0.2 mmol, two equivalent to chloromethyl groups on the PS copolymer), and NaH (0.020 g, 0.5 mmol) were added into anhydrous THF (1.5 mL). The mixture was stirred for 48 h at room temperature under argon. The reaction mixture was filtered to remove the generated salt and residual base, and the filtrate was evaporated to dryness. The crude graft polymer was then dialyzed against DI water for three days and lyophilized to give a white powder (0.25 g). ^{1}H NMR (400 MHz, $CDCl_3$, δ) 7.10 (m, 12.56 H), 6.55 (m, 6.30H), 4.48 (bs, 1H),3.75-3.25 (m, 33.65H), 1.41-2.15 (m, 12.95H), content of Ph-CH_2-PEG_{2000} (7.2%). GPC (THF, polystyrene standard): M_w 15700, M_n 13600, PDI 1.16.

Calculation of single probe mass

For inorganic nanoparticle: $M_{NPs} = xM_A+yM_B+zM_C...$ (1)

Ratio of mass content $= xM_A : yM_B : zM_C...$ (2)

$V_{NPs} = xV_A+yV_B+zV_C...$, means that $r_{NPs}^3 = xr_A^3+yr_B^3+zr_C^3...$ (3)

Where M_{NPs} is the single particle mass of inorganic nanoparticle; M_A, M_B, M_C... are the single atom mass of different element which doped in nanoparticle (can be obtained by molar mass to N_A-6.02×10^{23} of different element); x, y, z are the average number of different element atoms in single nanoparticle; V_{NPs} is the single particle volume of inorganic nanoparticle; V_A, V_B, V_C are the single atom volume of different element which doped in nanoparticle; r_{NPs} is the single particle semidiameter of inorganic nanoparticle (can be determined by TEM and DLS characterization, the morphology of nanoparticles are seen as sphere); r_A, r_B, r_C are the single atom semidiameter of different element which doped in nanoparticle. According to (2) and (3), the value of x, y, z can be achieved, then the value of M_{NPs} can be obtained.

For organic nanoparticle: $M_{ONPs} \approx \rho V_{ONPs} = \rho 4\pi r^3_{ONPs}/3$ (4)

Where M_{ONPs} is the single particle mass of organic nanoparticle; ρ is the density of organic nanoparticles (the value of ρ is nearly to the density of water, 1.00 g/mL); V_{ONPs} is the single particle volume of organic nanoparticle; r_{ONPs} is the single particle semidiameter of organic nanoparticle (can be determined by TEM and DLS characterization, the morphology of nanoparticles are seen as sphere).

Calculation of the dye contents in organic nanoparticles

A certain volume of aqueous solution contained organic nanoparticles are dried. The weight of the nanoparticles powder is measured for mass concentration calculation, termed C_{NPs}. The nanoparticles powder is then dissolved in a certain volume of tetrahydrofuran (THF), and BBTD are extracted in THF. The absorbance of BBTD at 760 nm in THF was measured. According to the extinction coefficient of BBTD in THF at 760 nm (1.5×10^4 M^{-1}cm^{-1}), the

molar concentration of BBTD ($C_{dye\text{-}molar}$) in THF can be obtained. Then the dye content of different organic nanoparticles can be calculated by following equation:

$$Dye\ content = \frac{m_{dye}}{m_{NPs}} = \frac{M_{dye}C_{dye-molar}}{C_{NPs-mass}} \quad (5)$$

Where m_{dye} is the mass of BBTD in THF solution; m_{NPs} is the mass of the organic nanoparticles in THF solution; M_{dye} is the molar mass of BBTD; $C_{dye\text{-}molar}$ is the molar concentration of BBTD in THF solution; $C_{NPs\text{-}mass}$ is the mass concentration of organic nanoparticles in THF solution (according to the C_{NPs}).

Calculation of single probe brightness

$$Single\ probe\ brightness = \frac{\varepsilon_{probe}\emptyset_{probe}}{N_A} \approx \frac{N_{dye}\varepsilon_{dye}\emptyset_{probe}}{6.02 \times 10^{23}} \quad (6)$$

Where ε_{probe} is the extinction coefficient of a single luminescent probe; $\emptyset_{probe}$ is the luminescence quantum yield of luminescent probe; when the probe is an organic nanoparticle, N_{dye} is the number of dyes in a single luminescent organic nanoprobe; ε_{dye} is the extinction coefficient of a single dye; N_A is the avogadro's number- 6.02×10^{23} . For organic nanoparticles, when the dye contents and diameter of organic nanoparticles have been determined, N_{dye} can be calculated by following equation.

$$N_{dye} = \frac{[dye\ content]M_{NPs}}{M_{dye}} \quad (7)$$

Where M_{NPs} is the molar mass of organic nanoparticle; M_{dye} is the molar mass of doped dyes.

Fluorescence quantum yield measurement

To measure the quantum yield of NIR-II Pdots, the reference fluorophore is IR26 in DCE (QY = 0.5%), Ex = 730 nm. The quantum yield was calculated in the following manner.

$$\phi = \phi_{ref} \times (\boldsymbol{n}^2_{sample}/\boldsymbol{n}^2_{ref})(\boldsymbol{I}_{sample}/\boldsymbol{A}_{sample})(\boldsymbol{A}_{ref}/\boldsymbol{I}_{ref}) \quad (8)$$

Difference concentrations at or below OD 0.1 were measured and the integrated fluorescence was plotted against absorbance for every fluorescent molecular. Comparison of the slopes led to the determination of the quantum yield of NIR-II Pdots.

Cytotoxicity test

The *in vitro* cytotoxicity was measured using a standard methyl thiazolyl tetrazolium (MTT, Sigma Aldrich) assay in SKOV3 and A2780 cell lines. Briefly, cells growing in log phase were seeded into 96-well cell culture plate at 1×10^4/well. NIR-II Pdots and NIR-Pdots-GnRH was added to the wells of the treatment group at concentrations of 0, 10, 100, 500, 1000 μg/mL. For the negative control group, 1 μL/well solvent was diluted in DMEM with the final concentration of 1 %. The cells were incubated for 24 h or 72 h at 37 °C under 5 % CO_2. The combined MTT/PBS solution was added to each well of the 96-well assay plate and incubated for an additional 4 h. After removal of the culture solution, 200 μL DMSO was added to each well, shaking for 10 min at shaking table. An enzyme-linked immunosorbent assay (ELISA) reader was used to measure the OD570 (absorbance value) of each well referenced at 490 nm. The following formula was used to calculate the viability of cell growth:

Viability (%) = (mean of absorbance value of treatment group / mean of absorbance value of control) × 100

Table S1. Photophysical properties of the reported NIR-II luminescent probes.

**NIR-II Dyes**	**Abs (nm)**	**Em (nm)**	**QY (%)**	**Extinction coefficient ($M^{-1}cm^{-1}$)**	**Extinction coefficient ($Lg^{-1}cm^{-1}$)**	**Dye contents**	**Brightness of single probe ×100N_A**	**Size (nm)**	**Ref.**
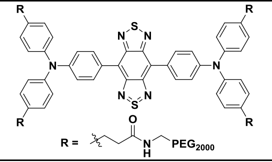	720	1055	0.3 H_2O	ND	ND	100%	ND	ND	**1**
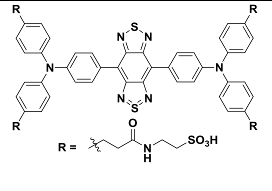	738<br>748<br>748	1055<br>1000<br>1000	0.1 H_2O<br>4.8 FBS<br>10.8 FBS -HT	8.5×10^3<br>9.1×10^3<br>9.8×10^3	6.1<br>0.068<br>0.07	100%<br>0.2%<br>0.2%	8.5×10^2<br>4.4×10^4<br>1.06×10^5	ND	**2**
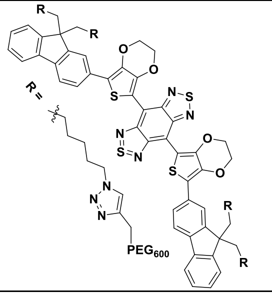	780	1047	2.0 H_2O	5.7×10^3	2.6	100%	1.14×10^4	4.9	**3**
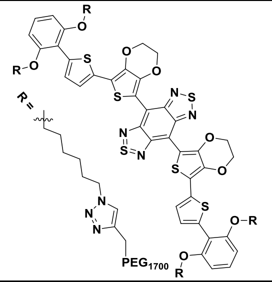	830	1071	0.7 H_2O	ND	ND	100%	ND	3.6	**4**
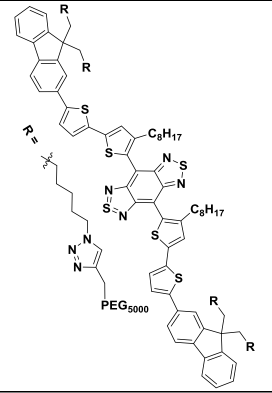	733	1048	5.3 H_2O	5.0×10^3	0.4	100%	2.65×10^4	ND	**5**
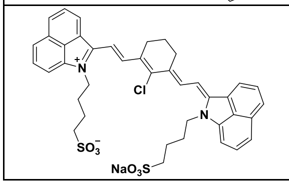	1046	1080	5.9 FBS	3.0×10^4	0.22	10.3%	1.77×10^5	ND	**6**

NIR-II ODNPs	Abs (nm)	Em (nm)	QY (%)	Extinction coefficient ($M^{-1}cm^{-1}$)	Extinction coefficient ($Lg^{-1}cm^{-1}$)	Dye contents	Brightness of single probe $\times 100N_A$	Size (nm)	Ref.
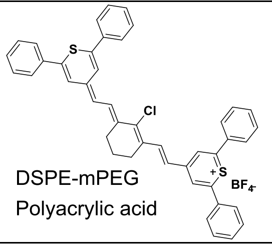	780 and 1047	920 and 1064	1.8 H_2O	ND	ND	0.8% Mol2%		5.8	**7**
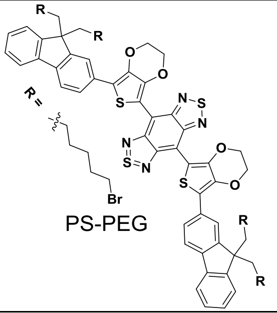	774	1010	16.5 H_2O	1* 1.9×10^4	0.0026	0.2%	1* 3.14×10^5	12	**8**
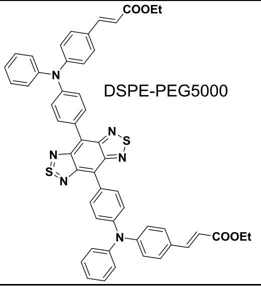	680	990	11.1 H_2O	1405* 7.4×10^3	0.12	14.3%	1405* 8.2×10^4	23.5	**9**
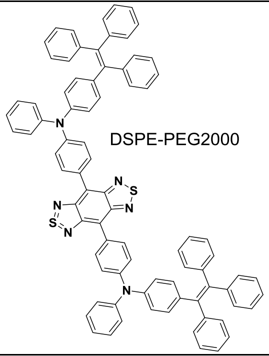	740	975	6.2 H_2O	2.0×10^8 N ≈ 5590	10.2	33.3%	1.2×10^9 N ≈ 5590	40	**10**

NIR-II ODNPs	Abs (nm)	Em (nm)	QY (%)	Extinction coefficient ($M^{-1}cm^{-1}$)	Extinction coefficient ($Lg^{-1}cm^{-1}$)	Dye contents	Brightness of single probe $\times 100N_A$	Size (nm)	Ref.
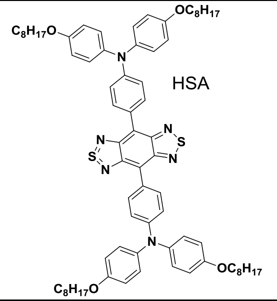	710	1050	1.45<br>H_2O	21800*<br>2.3×10^4	1.21	6.2%	21800*<br>3.3×10^4	110	**11**
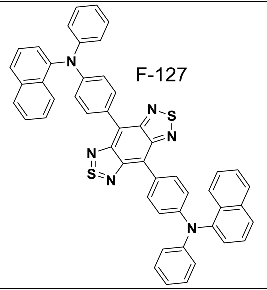	700	950	1.8<br>H_2O	1990*<br>1.3×10^4	1.26	7.7%	1990*<br>2.3×10^4	40	**12**
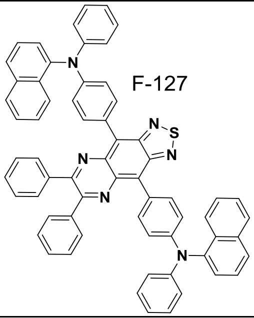	630	810<br>>900	13.9<br>2.8<br>H_2O	1030*<br>2×10^4	1.66	7.7%	1030*<br>5.6×10^4	33	**13**
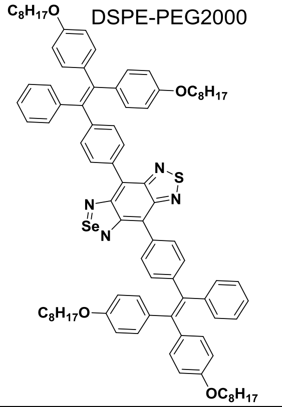	715	897	5.8<br>H_2O	2890*<br>3×10^4	7.03	33.3%	2890*<br>1.7×10^5	34	**14**

NIR-II CPs	Abs (nm)	Em (nm)	QY (%)	Extinction coefficient ($M^{-1}cm^{-1}$)	Extinction coefficient ($Lg^{-1}cm^{-1}$)	Lumin-ophor contents	Brightness of single probe $\times 100N_A$	Size (nm)	Ref.
DSPE-PEG5000<br>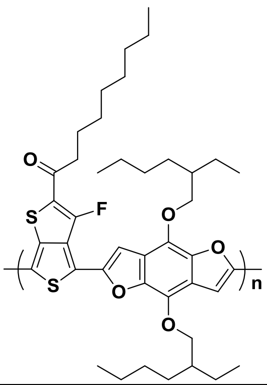	654	1047	1.7<br>H_2O	1*<br>4.9×10^5	0.23	0.75%	1*<br>8.3×10^5	6	**15**
PS-PEG<br>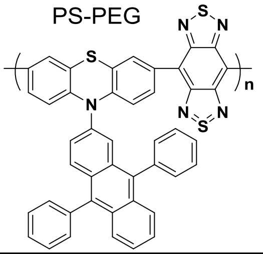	759	1083	1.7<br>H_2O	ND	ND	80%	ND	16	**16**
**NIR-II QDs**									
Ag_2S	600	1058	15.5<br>H_2O	1.3×10^6	2.73	~100%	2.0×10^7	5.4	**17**
$InAs(CdSe)_1(ZnSe)_3$	900	1080	31<br>H_2O	1.2×10^5	ND	100%	3.7×10^6	4.3	**18**
PbS/CdS	809	1650	22<br>H_2O	2.0×10^5	ND	100%	4.4×10^6	7	**19**
**NIR-II LnNPs**									
$NaYbF_4$:2%Er,2%Ce<br>@$NaYF_4$	980	1550	2.7	1.3×10^3	1.3×10^{-5}	100%	3.5×10^3	22<br>7	**20**
$NaYF_4$:7%Yb,60%Nd<br>@CaF_2	808	980	20.7	2.06	7.8×10^{-6}	100%	42.6	6<br>3	**21**
**NIR-II Pdots**	**710**	**1020**	**5.4**<br>H_2O	**4.1×10^7**	**16.2**	**26.3%**	**2.2×10^8**	**20**	**This work**

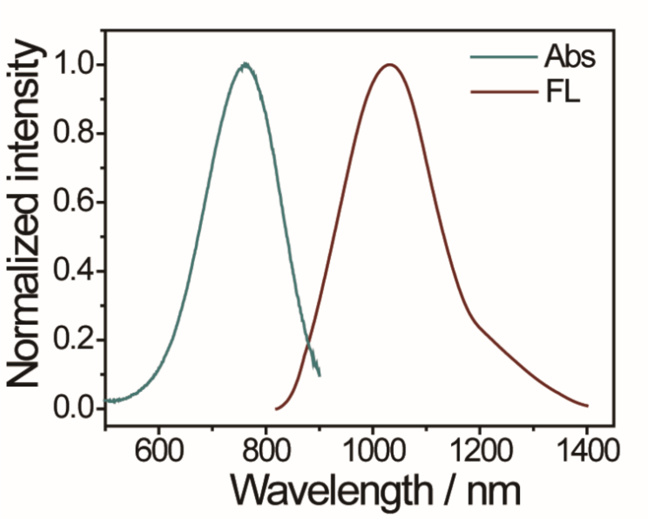

Figure S1. Absorption and emission spectra of BBTD in toluene.

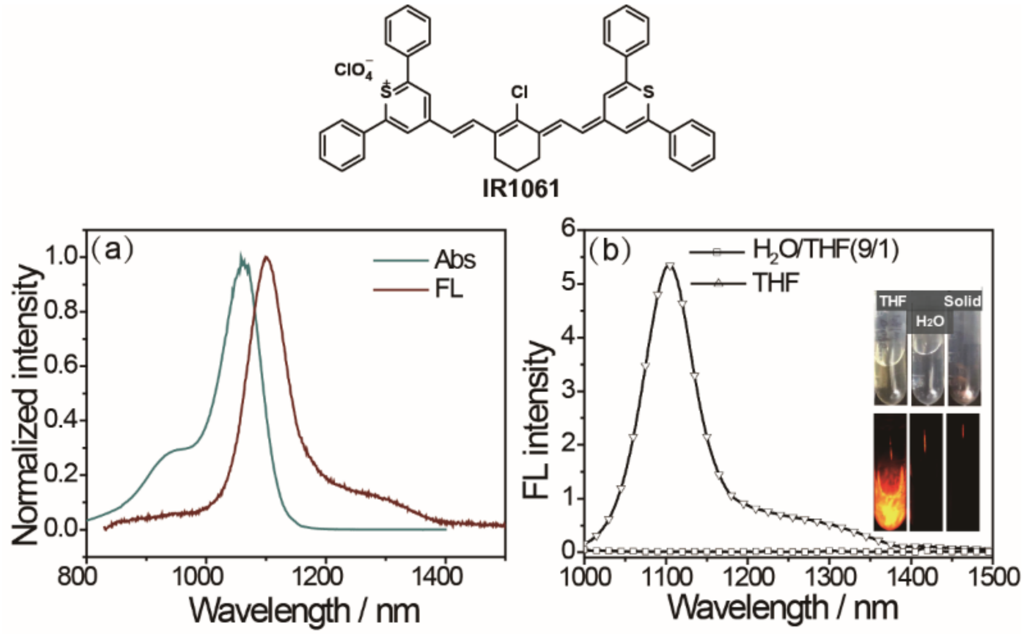

Figure S2. (a) Absorption and emission spectra of IR1061 in dichloromethane (DCM). (b) Fluorescence spectra of IR1061 in tetrahydrofuran (THF) and THF/H_2O mixture (1/9). Inset: NIR-II fluorescence images of BBTD in THF solution (left), THF/H_2O mixture (1/9) (medium) and solid powder (right) under 730 nm illumination (20 mW cm^{-2}), respectively. Emission signal collection: 1000 nm long-pass filter (1000LP), exposure time: 20 ms.

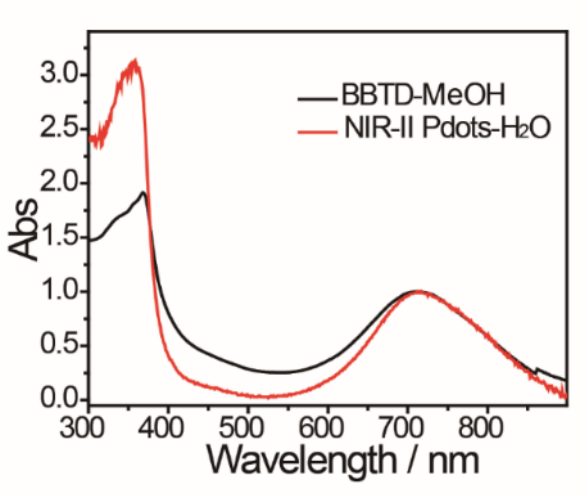

Figure S3. Absorption spectra of BBTD in methanol (MeOH) and NIR-II Pdots in water.

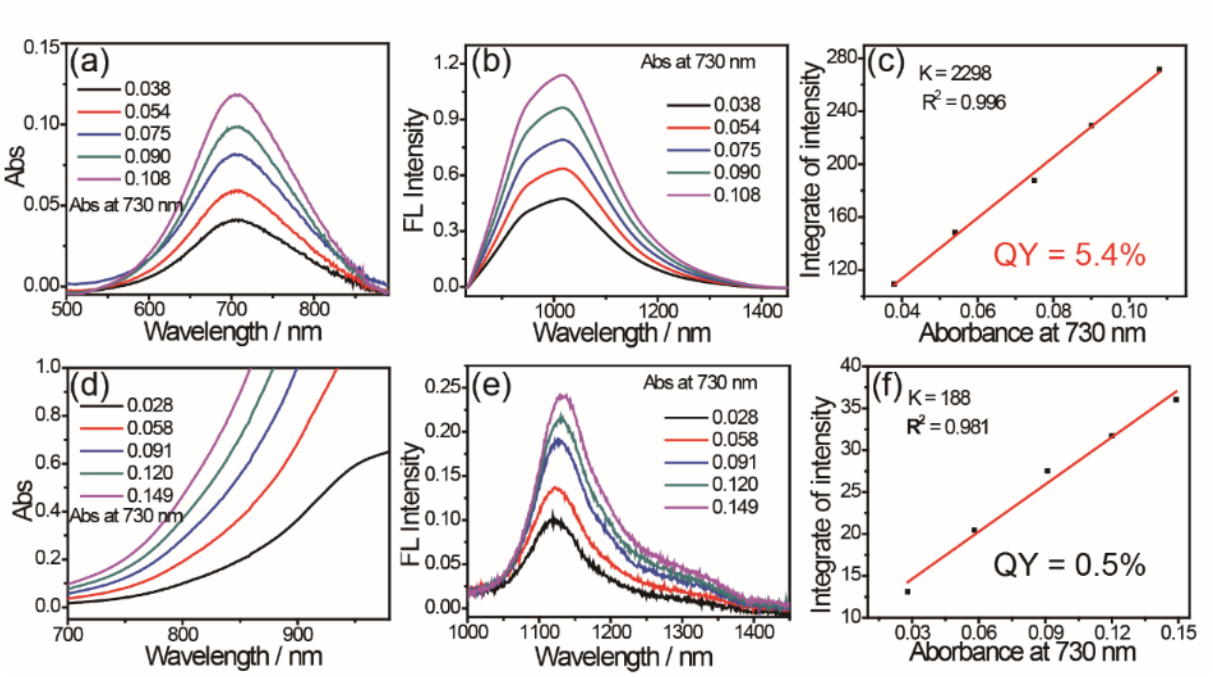

Figure S4. Fluorescence quantum yield measurement of the NIR-II Pdots. (a) Absorption spectra of different concentrations of NIR-II Pdots in water. (b) Corresponding fluorescence spectra of different concentrations of NIR-II Pdots in water shown in a), excitation: 730 nm. (c) Integrated NIR-II fluorescence intensity plotted as a function of absorbance at 730 nm for NIR-II Pdots solutions based on the measurements in a) and b). The data was fitted into a linear function with a slope of 2298. (d) Absorption spectra of different concentrations of IR26 in 1,2-dichloroethane (DCE). (e) Corresponding fluorescence spectra of different concentrations of IR26 in DCE shown in d), excitation: 730 nm. (f) Integrated NIR-II fluorescence intensity plotted as a function of absorbance at 730 nm for IR26 solutions based on the measurements in d) and e). The data was fitted into a linear function with a slope of 188.

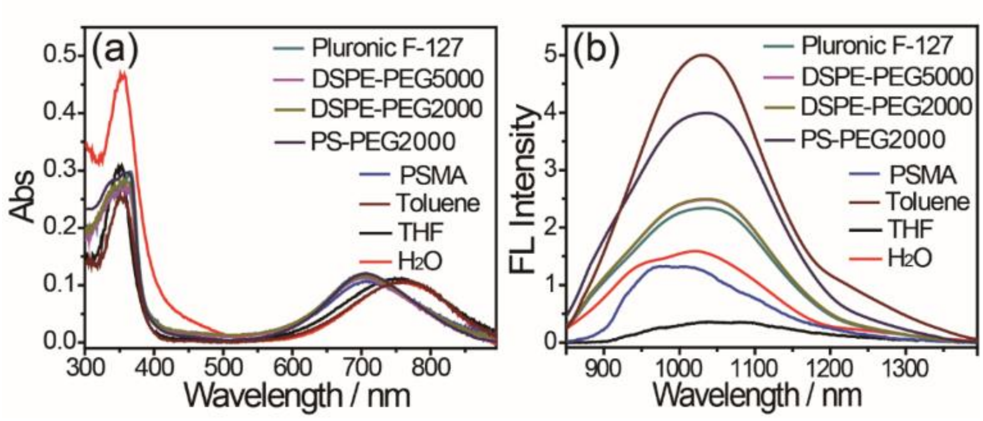

Figure S5. (a) Absorption and (b) fluorescence spectra of BBTD in different solvent and BBTD loaded by different polymer disperse in water.

Table S2. Characterizations of the nanoparticles that consisted by BBTD and different polymers.

Polymer	F-127	DSPE-PEG5000	DSPE-PEG2000	PS-PEG2000	PSMA
m_{Dye} %	10.2%	5.3%	9.1%	26.3%	16.5%
QY	3.42%	3.7%	3.7%	5.4%	1.89%
Diameter (PDI)	39 nm (0.214)	40 nm (0.152)	42 nm (0.174)	20 nm (0.124)	29 nm (0.112)

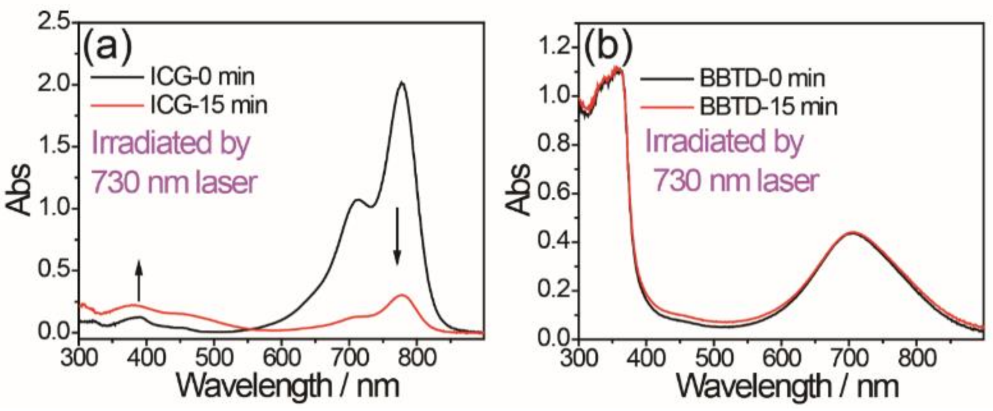

Figure S6. Photostability measurement of ICG and NIR-II Pdots under the irradiation of 730 nm laser in water. Absorption spectra of ICG (a) and NIR-II Pdots (b) before and after irradiation.

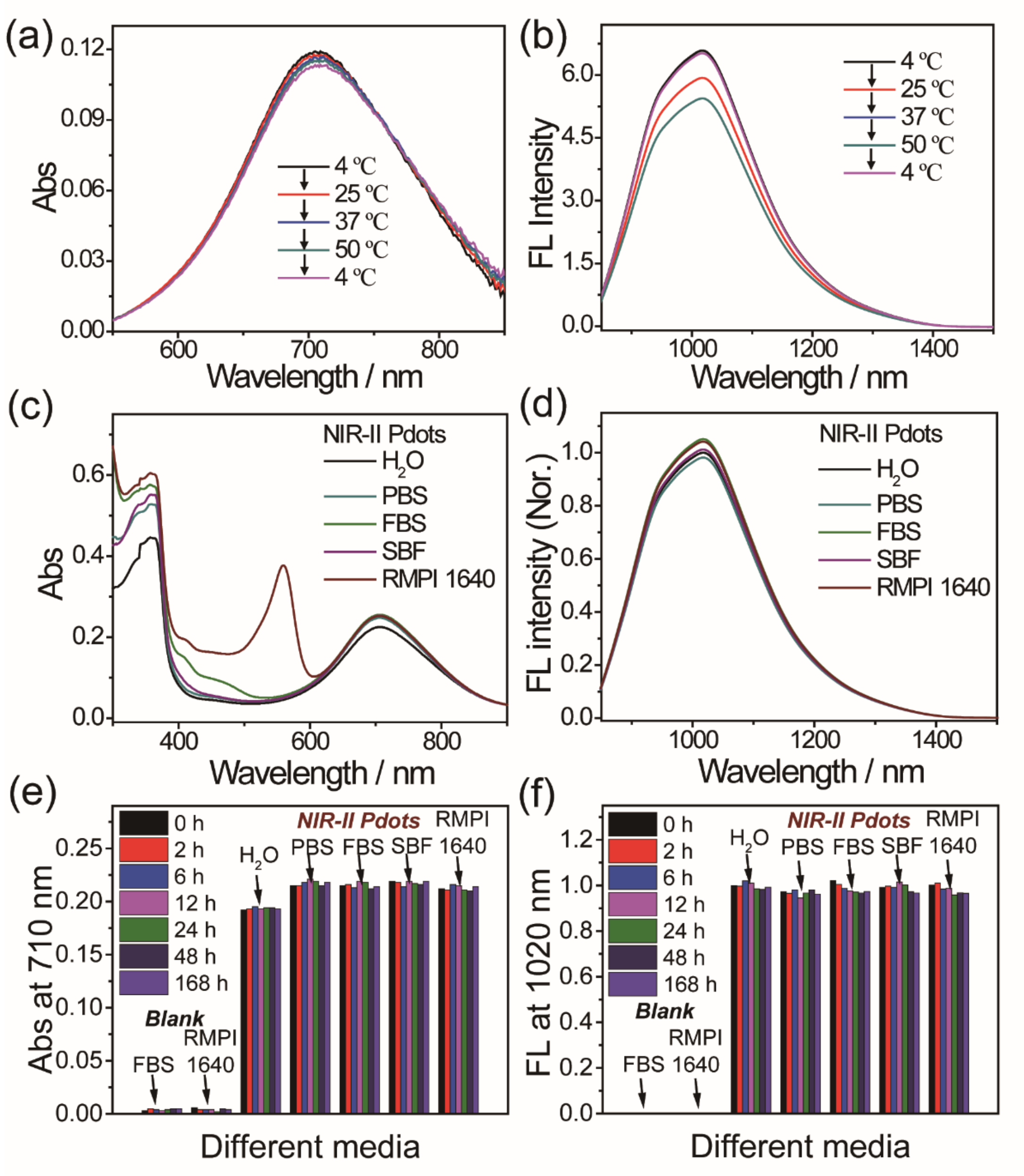

Figure S7. Photostability measurement of NIR-II Pdots at different temperature and different medias. Absorption (a) and fluorescence (b) spectra of NIR-II Pdots under different temperature. Absorption (c) and fluorescence (d) spectra of NIR-II Pdots under different medias. Time-dependent absorbance at 710 nm (e) and time-dependent fluorescence intensity at 1020 nm (f) of NIR-II Pdots in different medias. *Blank FBS*-10% fetal bovine serum (FBS) contained PBS solutions, *blank RMPI 1640*-10% FBS contained RMPI 1640 media, *NIR-II Pdots H_2O*-NIR-II Pdots in ultrapure water, *NIR-II Pdots PBS*-NIR-II Pdots in 1M phosphate buffer solutions (PBS, pH 7.4), *NIR-II Pdots FBS*-NIR-II Pdots in 10% FBS contained PBS solutions, *NIR-II Pdots SBF*-NIR-II Pdots in simulated body fluid (SBF) solutions, *NIR-II Pdots RMPI 1640*-NIR-II Pdots in 10% FBS contained RMPI 1640 media. Mass concentration of NIR-II Pdots is 0.02 mg/mL.

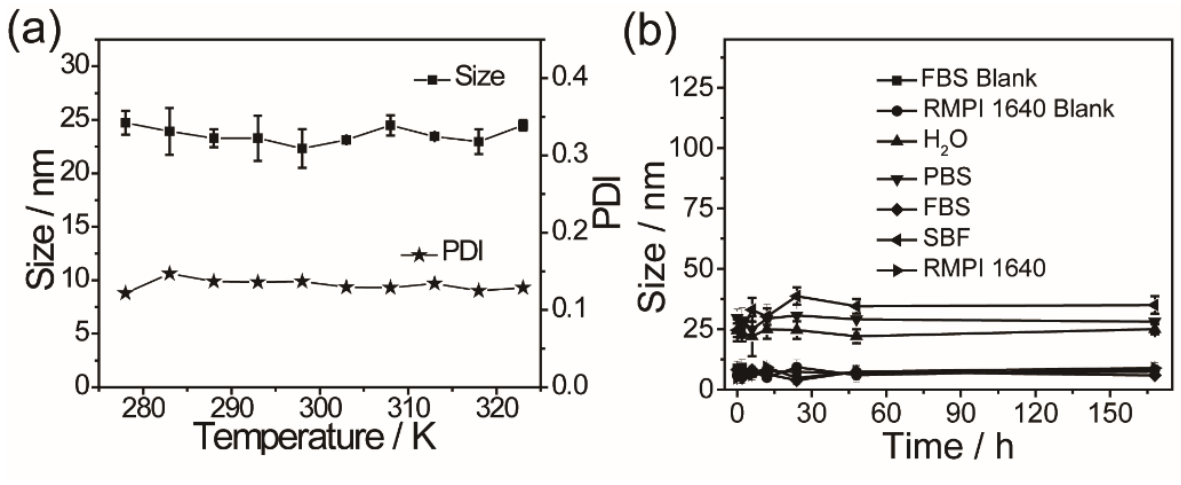

Figure S8. (a) Diameter measurement of NIR-II Pdots in H_2O at different temperature. (b) Diameter measurement of blank fetal bovine serum (FBS) contained PBS solutions (FBS content, 10%), blank serum contained RMPI 1640 media (contain 10% FBS) and NIR-II Pdots contained water, PBS, FBS (10%), simulated body fluid (SBF) solutions and RMPI 1640 media (contain 10% FBS) at different time point.

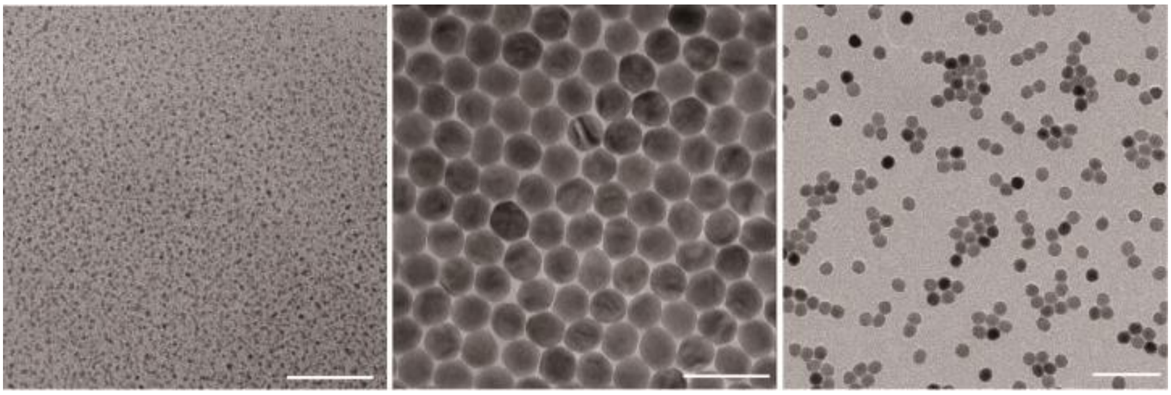

Figure S9. TEM of PbS QDs (left), $NaYF_4$-7%Yb-60%Nd (medium), $NaYF_4$-5%Nd (right). Scale bar-100 nm

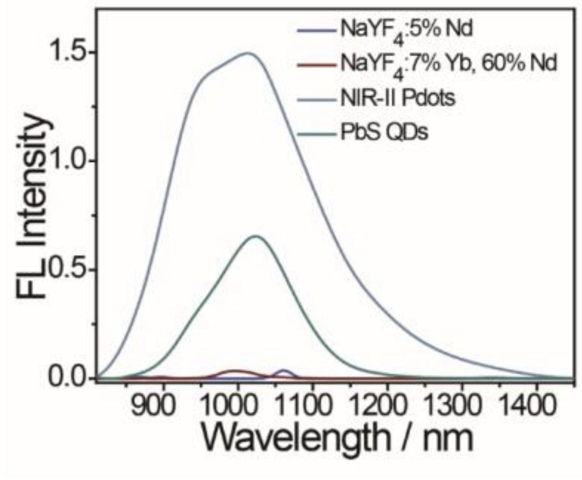

Figure S10. Fluorescence spectra of different kind of NIR-II emissive nanoparticles at an equal mass concentrations and excitation power. Solvent: water, mass concentrations: 0.05 mg/mL, excitation power: 5 mW cm^{-2}

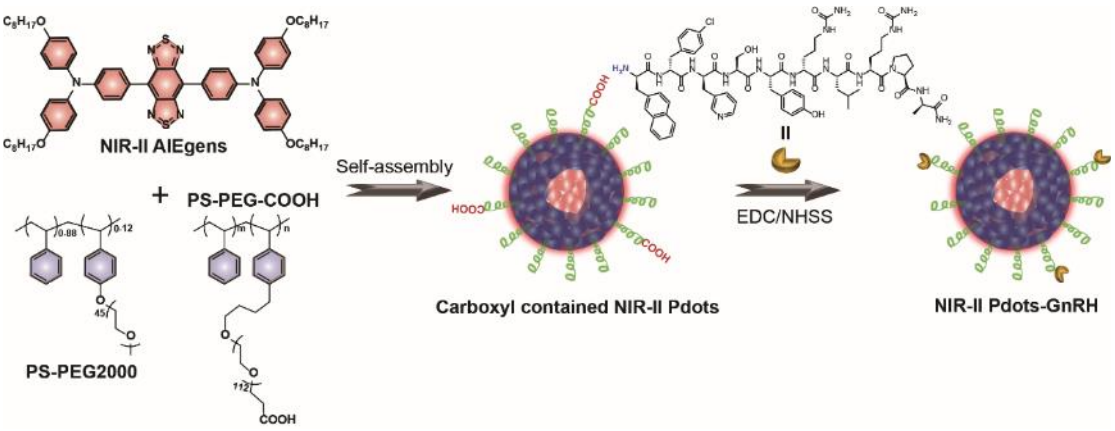

Scheme S2. Preparation procedure of NIR-II Pdots-GnRH**.**

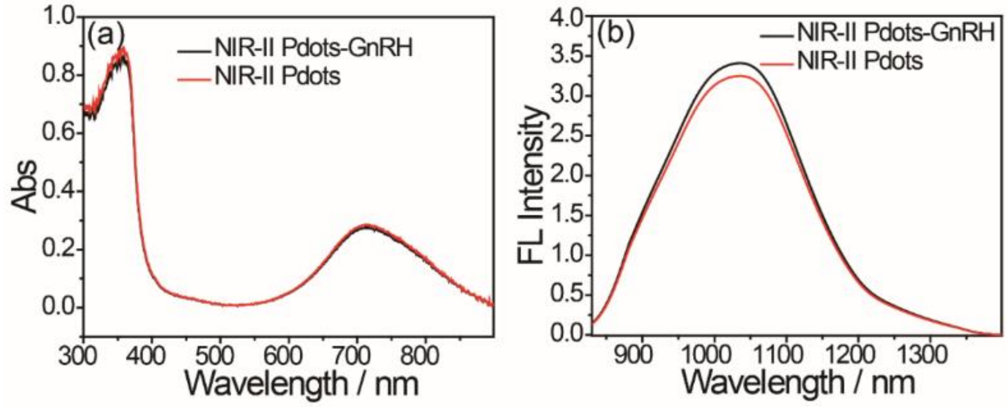

Figure S11. (a) Absorption and (b) fluorescence spectra of NIR-II Pdots and NIR-II Pdots-GnRH at equal mass concentrations. Mass concentration: 0.02 mg/mL.

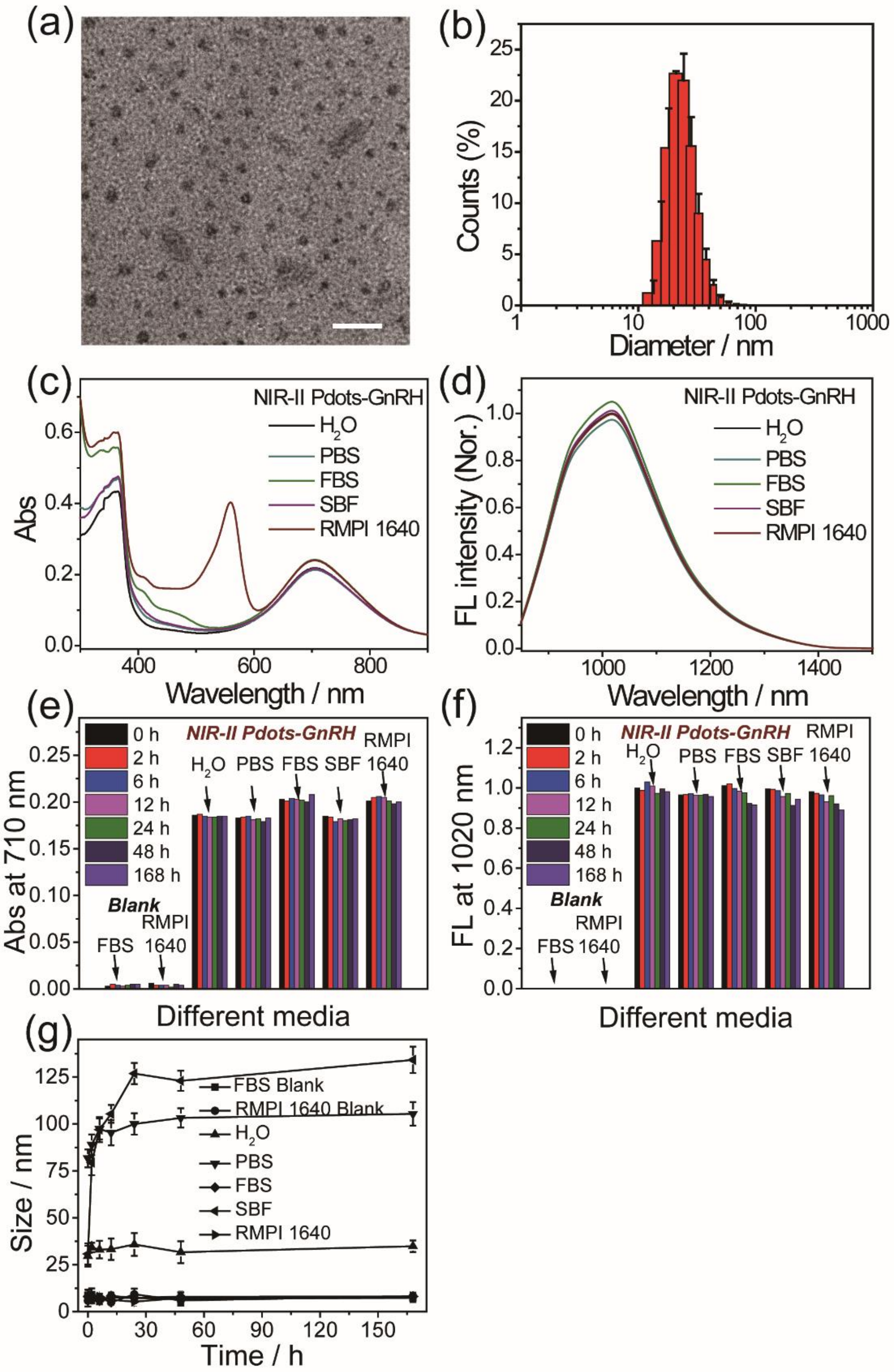

Figure S12. (a) TEM and (b) DLS characterization of NIR-II Pdots-GnRH. Scale bar: 100 nm. Absorption (c) and fluorescence (d) spectra of NIR-II Pdots-GnRH under different medias.

Time-dependent absorbance at 710 nm (e), time-dependent fluorescence intensity at 1020 nm (f) and time-dependent diameter (g) of NIR-II Pdots-GnRH in different medias. *Blank FBS*-10% fetal bovine serum (FBS) contained PBS solutions, *blank RMPI 1640*-10% FBS contained RMPI 1640 media, *NIR-II Pdots-GnRH H_2O*-NIR-II Pdots-GnRH in ultrapure water, *NIR-II Pdots-GnRH PBS*-NIR-II Pdots-GnRH in 1M phosphate buffer solutions (PBS, pH 7.4), *NIR-II Pdots-GnRH FBS*-NIR-II Pdots-GnRH in 10% FBS contained PBS solutions, *NIR-II Pdots-GnRH SBF*-NIR-II Pdots-GnRH in simulated body fluid (SBF) solutions, *NIR-II Pdots-GnRH RMPI 1640*-NIR-II Pdots-GnRH in 10% FBS contained RMPI 1640 media. Mass concentration of NIR-II Pdots-GnRH is 0.02 mg/mL.

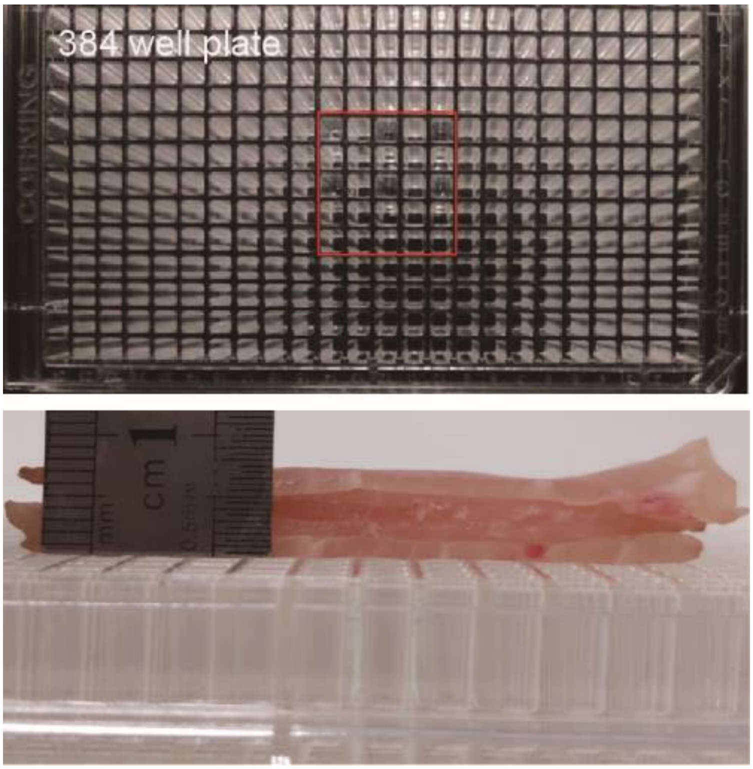

Figure S13. Study of penetration depth and detection sensitivity for NIR-II fluorescence imaging (1100 long-pass filter, 200 ms) under 730 nm irradiation (25 mW/cm^2).

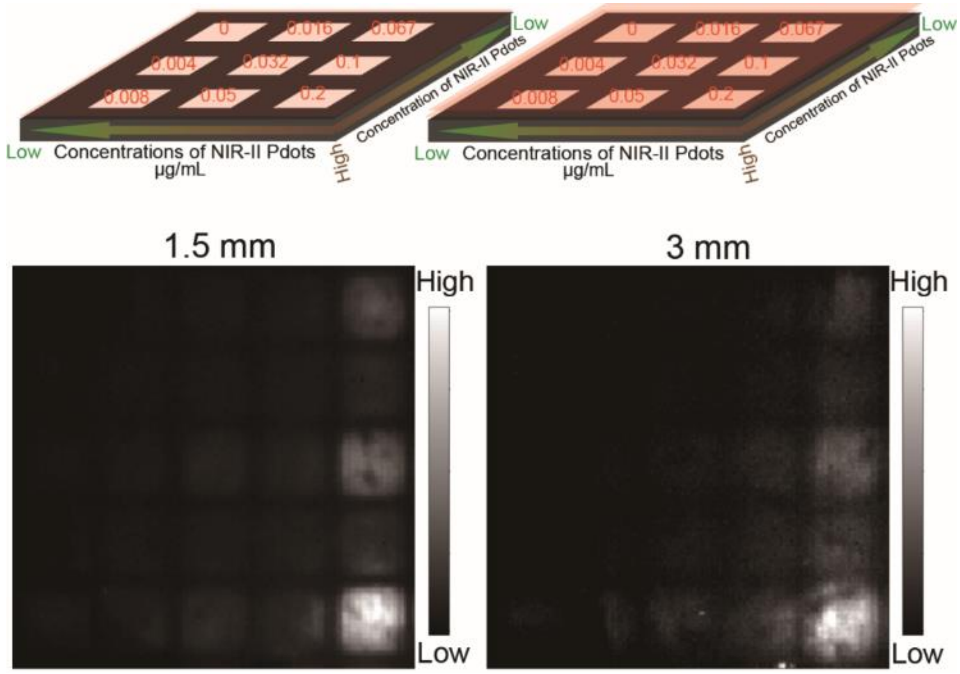

Figure S14. Simplified diagram depicting the experimental setup of the *in vitro* experiment with the cover of 1.5 mm (left) and 3 mm (right) pork tissue and the corresponding NIR-II fluorescence images.

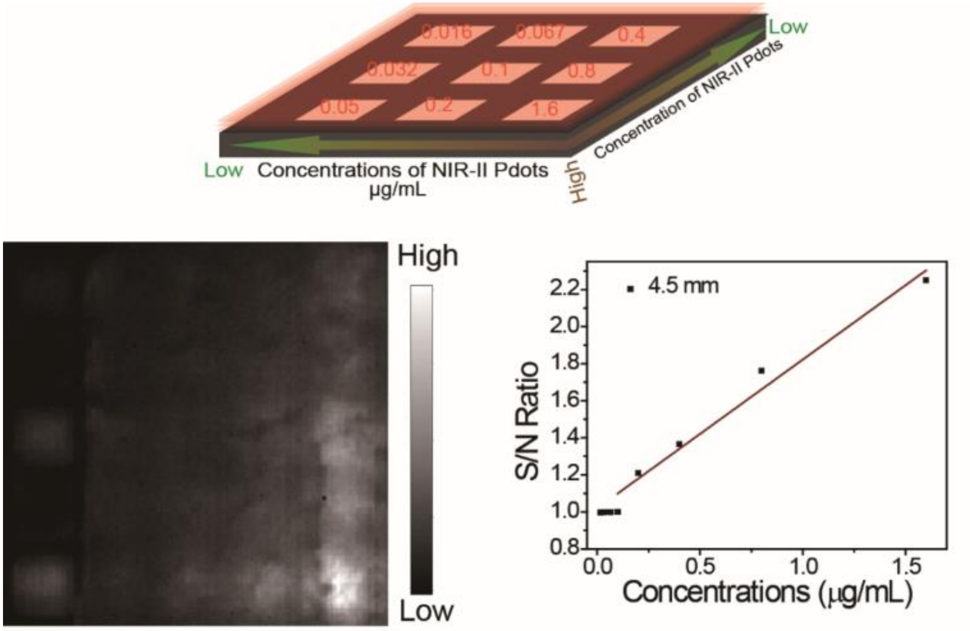

Figure S15. Simplified diagram depicting the experimental setup of the *in vitro* experiment with the cover of 4.5 mm pork tissue, the corresponding NIR-II fluorescence images and corresponding plots of the S/N ratio value as a function of the concentrations of NIR-II Pdots under the cover of 4.5 mm pork tissue.

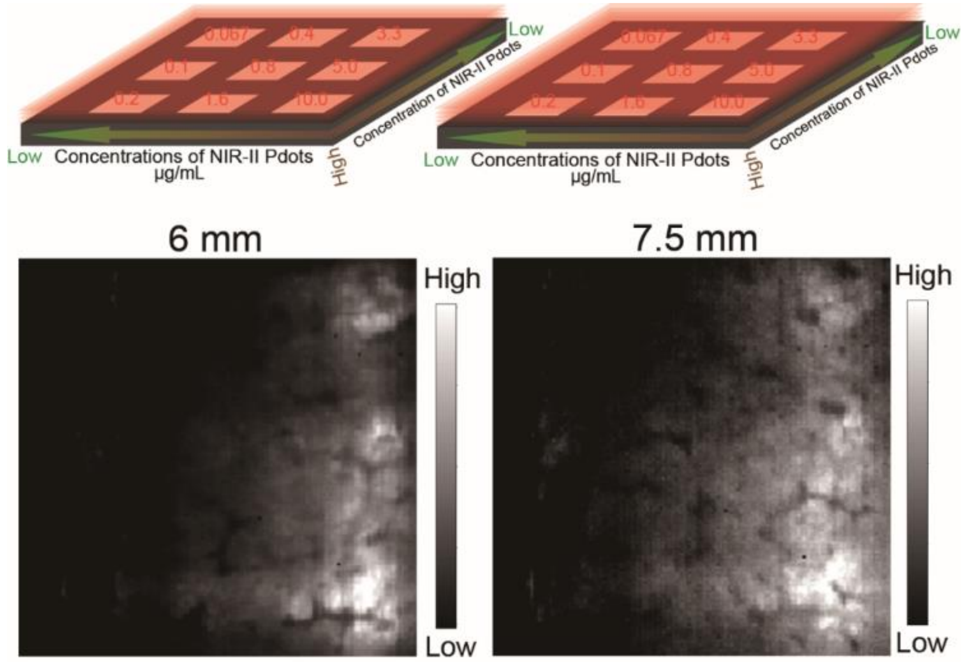

Figure S16. Simplified diagram depicting the experimental setup of the *in vitro* experiment with the cover of 6 mm (left) and 7.5 mm (right) pork tissue and the corresponding NIR-II fluorescence images.

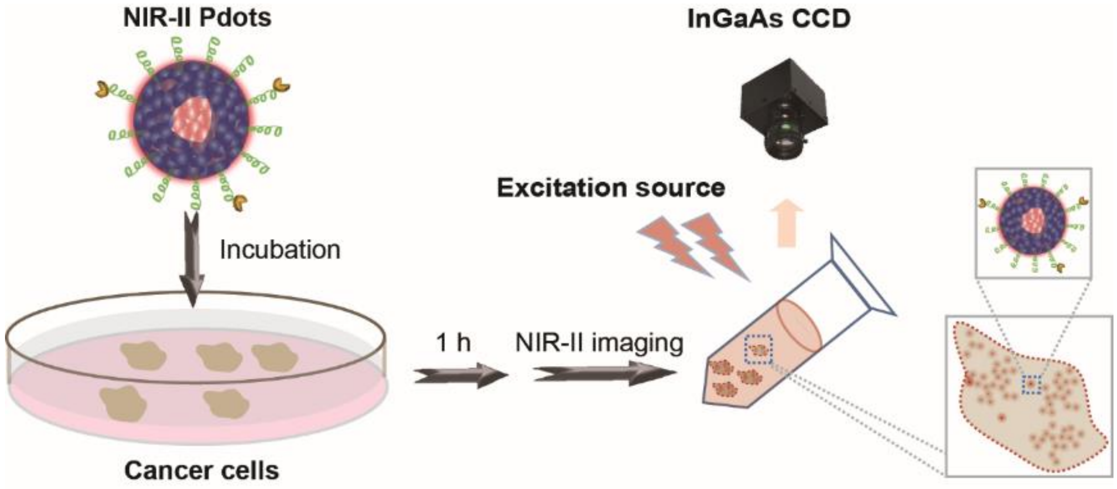

Figure S17. Illustration of the procedure of NIR-II fluorescence imaging of NIR-II Pdots and NIR-II Pdots-GnRH ovarian cancer cells at different concentrations.

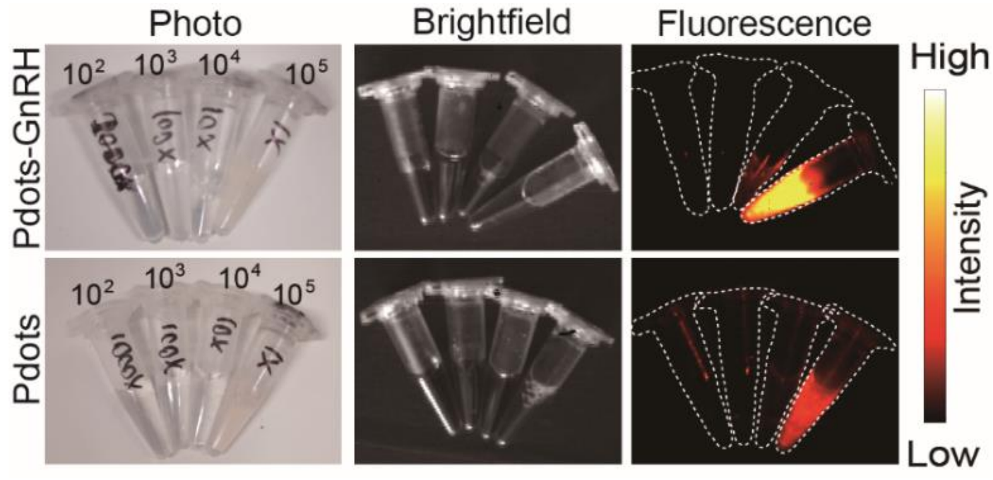

Figure S18. Photos, brightfield and NIR-II fluorescent images of NIR-II Pdots and NIR-II Pdots-GnRH labeled SKOV3 at different concentrations (10^2-10^5 cells/mL). Excitation: 730 nm; collected signal: 1100LP, 200 ms

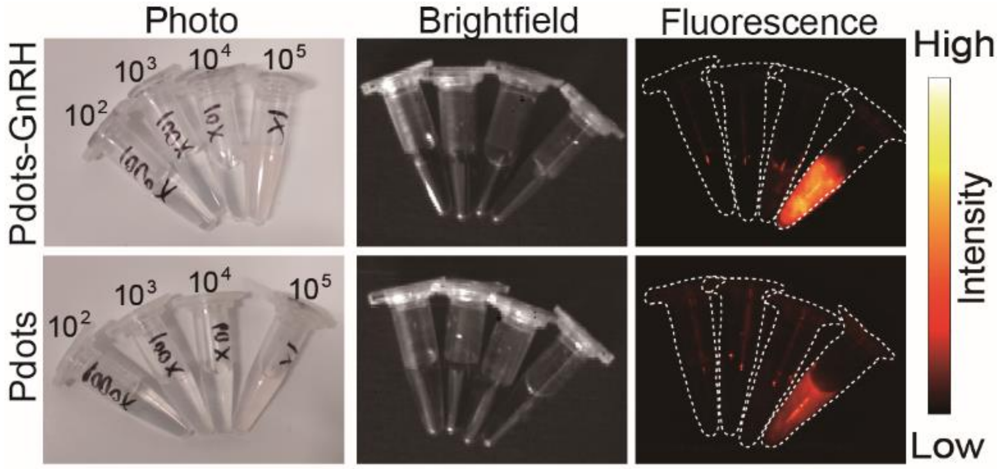

Figure S19. Photos, brightfield and NIR-II fluorescent images of NIR-II Pdots and NIR-II Pdots-GnRH labeled A2780 at different concentrations (10^2-10^5 cells/mL). Excitation: 730 nm; collected signal: 1100LP, 200 ms.

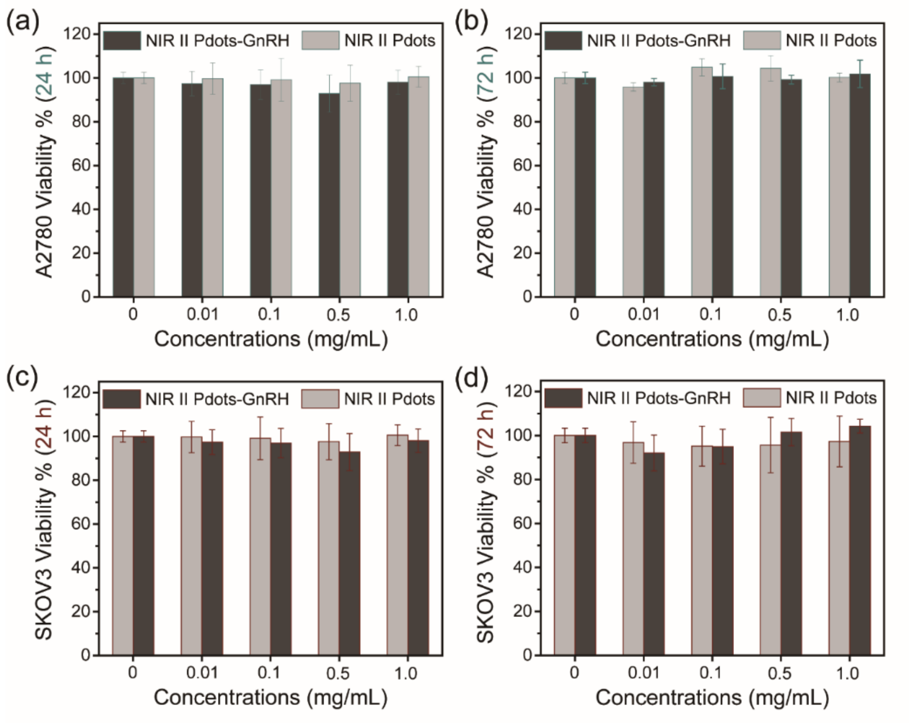

Figure S20. Cytotoxicity evaluation of NIR-II Pdots and NIR-II Pdots-GnRH over A2780 (a, b) and SKOV3 (c, d) cell lines with different incubation time (24 h and 72 h).

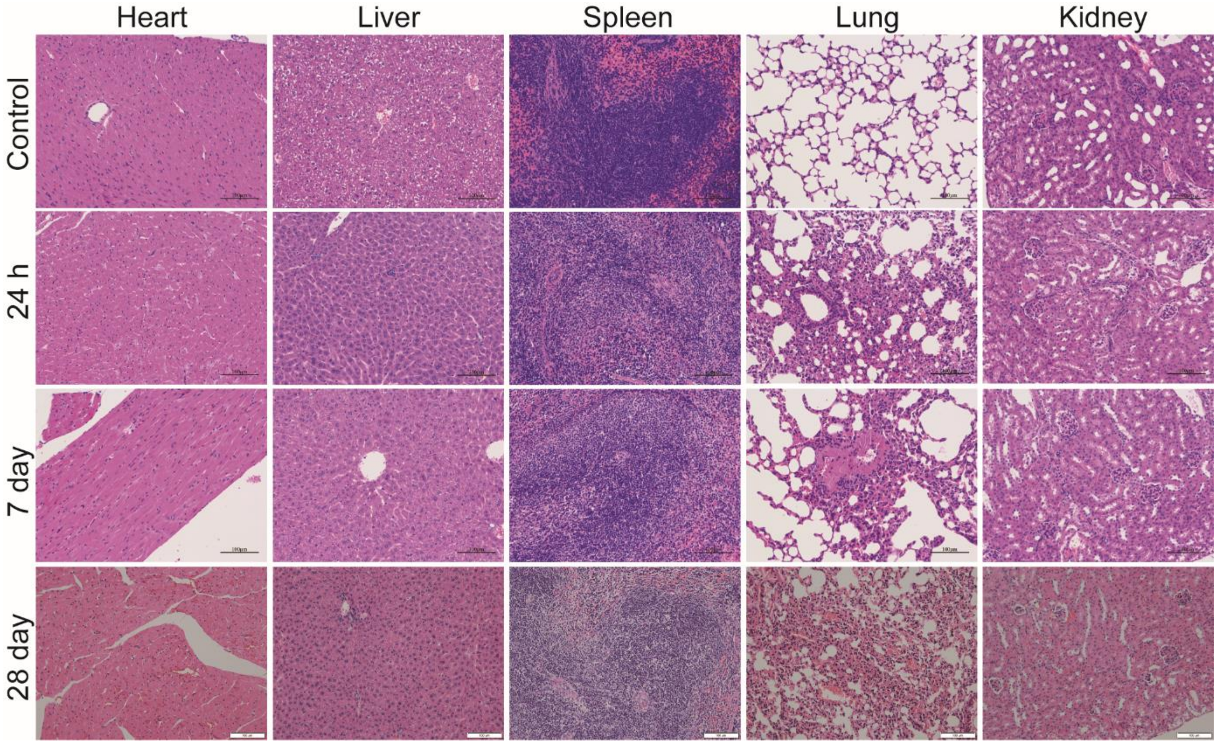

Figure S21. H&E-stained tissue sections of the heart, liver, spleen, lung and kidney harvested from mice after intravenous injection with PBS (control) or NIR-II Pdots-GnRH (1 mg/mL, 200 μL). Bar: 100 μm

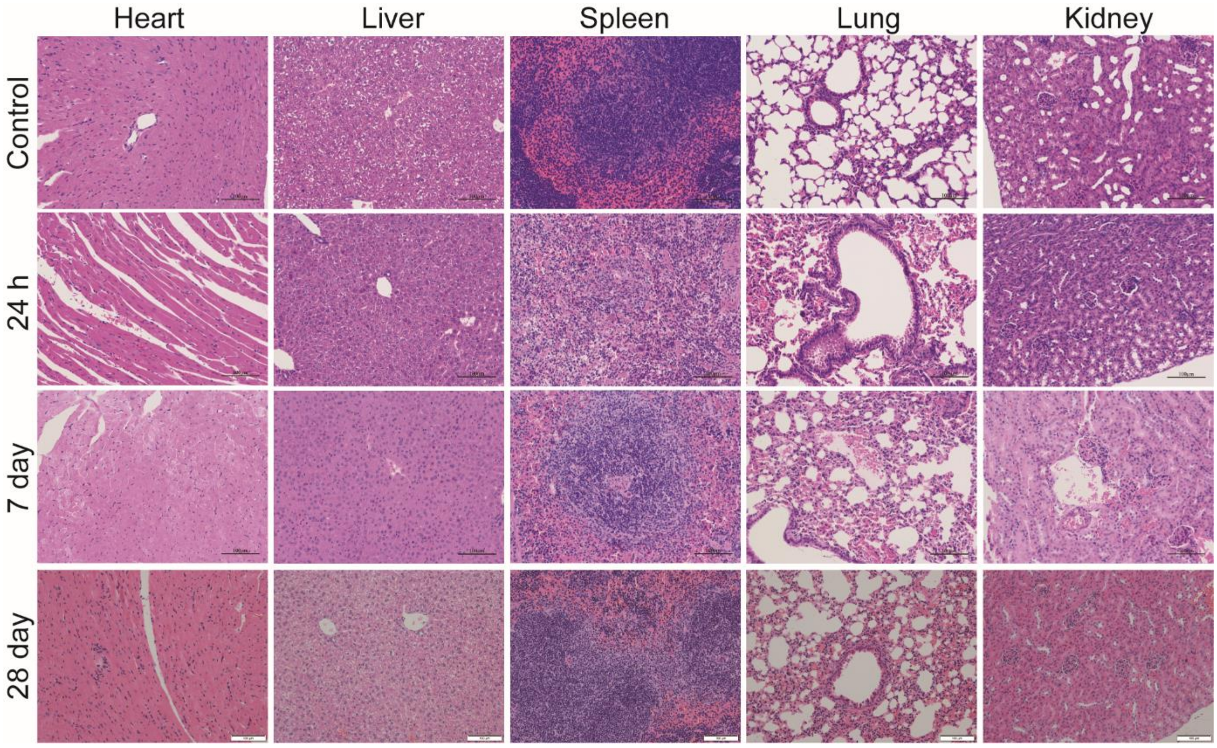

Figure S22. H&E-stained tissue sections of the heart, liver, spleen, lung and kidney harvested from mice after intravenous injection with PBS (control) or NIR-II Pdots (1 mg/mL, 200 μL). Bar: 100 μm.

Table S2. Blood chemistry and hematologic analysis of mice [a]

Item	Reference range	Control	1 Day		1 week		4 weeks	
			NIR-II Pdots-GnRH	NIR-II Pdots	NIR-II Pdots-GnRH	NIR-II Pdots	NIR-II Pdots-GnRH	NIR-II Pdots
WBC(10^9/L)	0.8-6.8	4.6±1.6	4.6±1.4	3.5±0.7	6.8±1.5	5.7±2.3	3.9±1.1	4.1±1.3
LYMPH (10^9/L)	0.7-5.7	3.0±1.3	3.6±1.4	2.5±0.7	5.2±1.4	3.6±1.2	3.2±0.8	3.4±1.1
MONO (10^9/L)	0.0-0.3	0.2±0.07	0.1±0	0.1±0.06	0.2±0.05	0.3±0.25	0.08±0.04	0.1±0.06
NEUT (10^9/L)	0.1-1.8	1.5±0.4	0.9±0.19	0.8±0.3	1.4±0.2	1.8±1.0	0.6±0.3	0.6±0.2
LYMPH (%)	55.8-90.6	63.0±5.6	75.4±8.4	70.7±10.5	75.2±4.4	66.4±10.2	82.3±3.2	81.8±3.1
MONO (%)	1.8-6.0	4.8±0.6	2.9±0.53	3.7±1.7	3.4±0.2	4.6±2.3	2.4±0.3	2.7±0.4
NEUT (%)	8.6-38.9	32.2±5.4	21.6±8.2	25.5±9.0	21.3±4.2	29±8.1	15.3±3.1	15.5±2.8
RBC (10^{12}/L)	6.36-9.42	9.5±1.9	9.9±1.2	9.5±0.5	10.4±0.3	10±0.6	11.3±2.1	9.4±1.1
HGB (g/L)	110-143	148±27	143±10.6	148.3±7.6	158.7±5.4	155.8±9.8	149.6±14.6	145.6±17.9
HCT (%)	34.6-44.6	45±10.6	48.1±5.5	45.8±5.2	51.4±2.1	49.6±3.9	54.3±10.7	45.1±5.6
MCV (fl)	48.2-58.3	46.9±3.1	48.7±1.2	48.3±2.0	49.7±0.8	49.3±1.3	48.3±0.5	48.3±0.7
MCH (pg)	15.8-19	15.6±0.5	14.6±1.5	15.5±0.2	15.3±0.2	15.4±0.4	13.8±2.9	15.5±0.17
MCHC (g/L)	302-353	336.2±33.1	300±32.6	323.7±16.8	308.3±2.8	314.6±13.7	286.8±61.4	322±2.5
RDW (%)	13-17	17.8±5.8	15.1±0.6	15.8±1.6	14.5±0.4	14.4±0.7	14.2±0.4	14.4±0.4
PLT (10^9/L)	450-1590	357.3±157.7	343.2±93.5	477±224	646±297.7	461.6±203	483.4±161	277.8±110
MPV (fl)	3.8-6.0	6.1±0.3	6.6±0.6	5.9±0.5	5.8±0.3	6.1±0.4	6.9±0.9	6.6±0.2
PDW (%)	15-17	16.7±0.5	16.9±0.4	16.7±0.3	17.5±0.2	17.5±0.6	17.5±0.4	18±0.7
PCT (%)	0.1-0.29	0.2±0.1	0.2±0.06	0.3±0.1	0.4±0.2	0.28±0.1	0.3±0.1	0.2±0.06
ALT (U/L)	10-116	87.8±25.4	94.7±25.7	99.7± 35.4	76.2±4.8	87.6±12.7	97.7±8.3	91.2±8.5
AST (U/L)	68-200	103.1±46.5	109.6±40.6	124.7±77.0	95.4±9.9	117.4±17.2	135.5±15.8	124.9±12.2
BUN (mg/dL)	13.6-25.8	22.7± 9.0	20.9±4.0	24.3±7.7	16.7±3.1	16.6±2.3	20.3±4.5	15.7±1.9
CREA (μM)	17.6-35.2	27.9± 5.2	47.4±9.4	33.5±14.9	36.3± 11.4	44.4±23.7	43.8±11.7	38.6±5.6

[a)] Blood or serum sample were collected from ICR mice (female, 4-6 weeks) at 1, 7 or 28 days dafter intravenous injection of NIR II Pdots and NIR II Pdots-GnRH (4 mg kg-1). PBS injected mice were used as control. Complete blood counts: Blood levels of White blood cells (WBC), Lymphocytes (LYMPH), Monocytes (MONO), Neutrophils (NEUT), Red blood cells (RBC), Hemoglobin (HGB), Hematocrit (HCT), Mean corpuscular volume (MCV), Mean corpuscular hemoglobin (MCH), Mean corpuscular hemoglobin concentration (MCHC), Red cell volume distribution width (RDW), Platelets (PLT), Mean platelet volume (MPV). Serum biochemistry data including blood urea nitrogen (BUN) levels, creatinine (CREA) and liver function markers such as Glutamic-pyruvic transaminase/ALT and Aspartate aminotransferase (AST) were also measured. Reference ranges of hematology data of healthy female ICR mice were obtained from Charles River Laboratories: (http://www.criver.com/). Data are presented as mean ± S.D. (n=5)

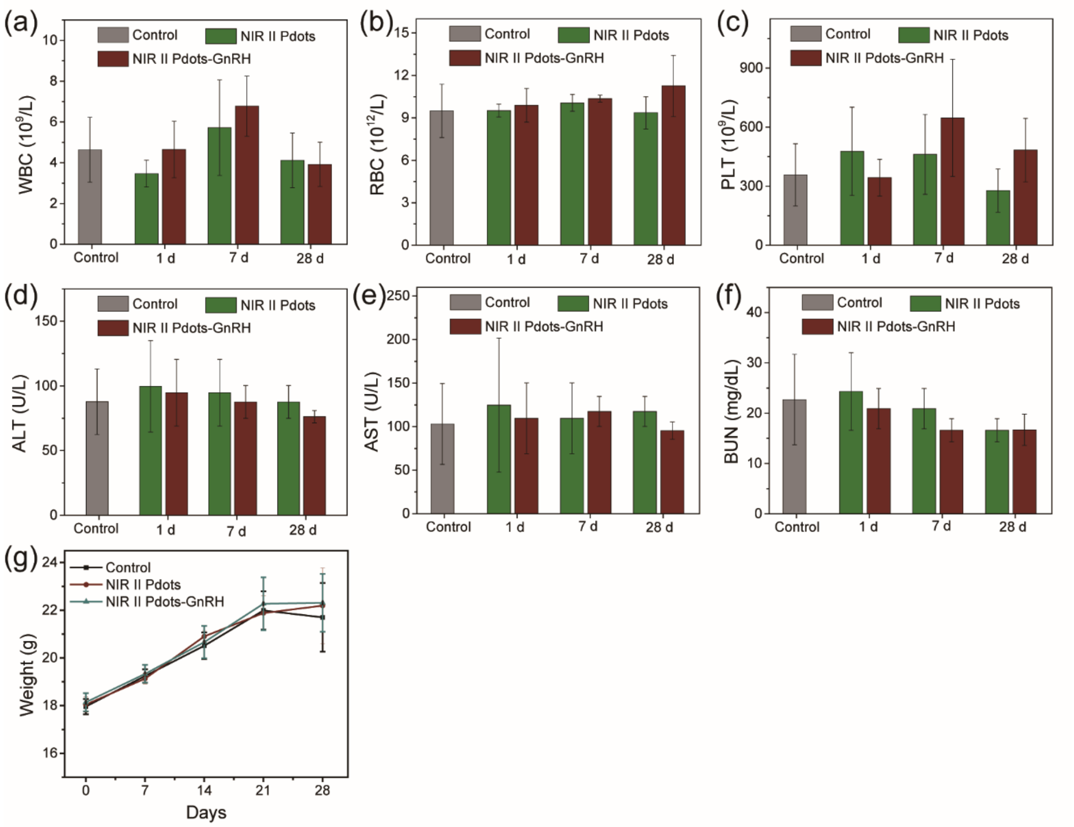

Figure S23. *In vivo* toxicity of NIR II Pdots and NIR II Pdots-GnRH. The results showed that the white blood cells (WBC) (a), red blood cells (RBC) (b), platelets (PLT) (c), alanine aminotransferase (ALT) (d), aspartate aminotransferase (AST) (e) and blood urea nitrogen (BUN) (f) were not significantly changed by Pdots treatment for 1 d, 7 d and 28 d. (g) The weight of PBS (control), NIR II Pdots and NIR II Pdots-GnRH treated mice. Bars show means ± SD; n = 5

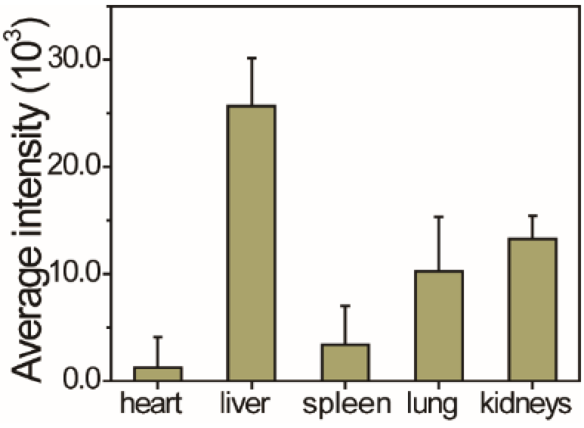

Figure S24. Quantitative results of fluorescence intensity for the ex vivo images of different organs shown in Figure 3c.

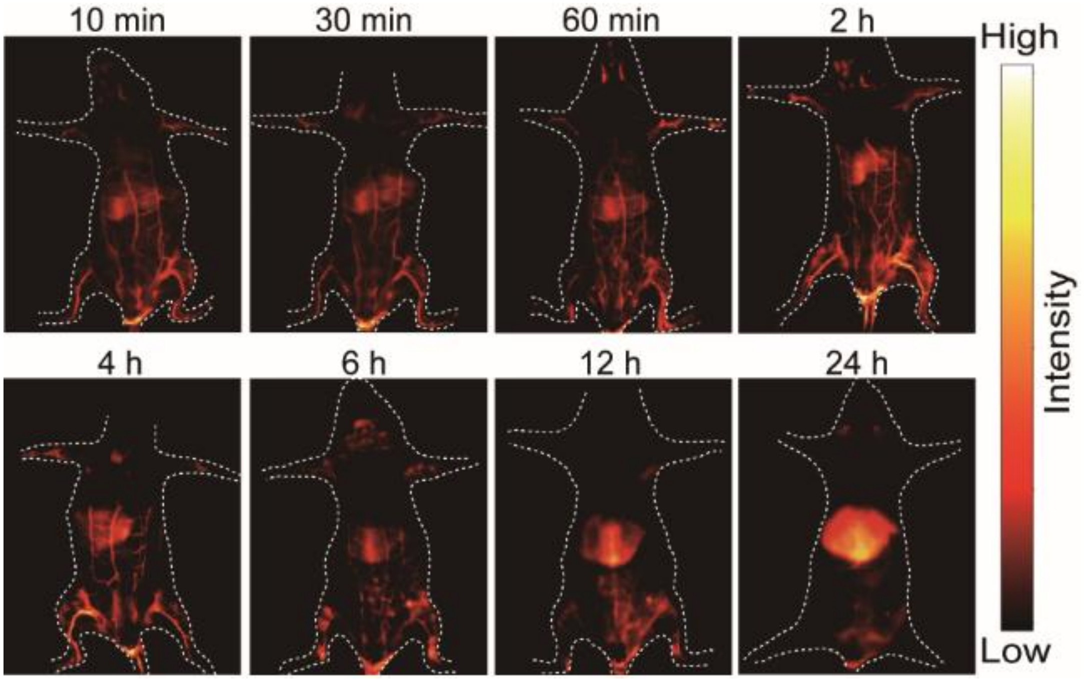

Figure S25. Time course of NIR-II fluorescence images of living mice injected with 200 μL (1 mg/mL) NIR-II Pdots-GnRH. Images were acquired at 10 min, 0.5 h, 1 h, 2 h, 4 h, 6 h, 12 h and 24 h pi, respectively.

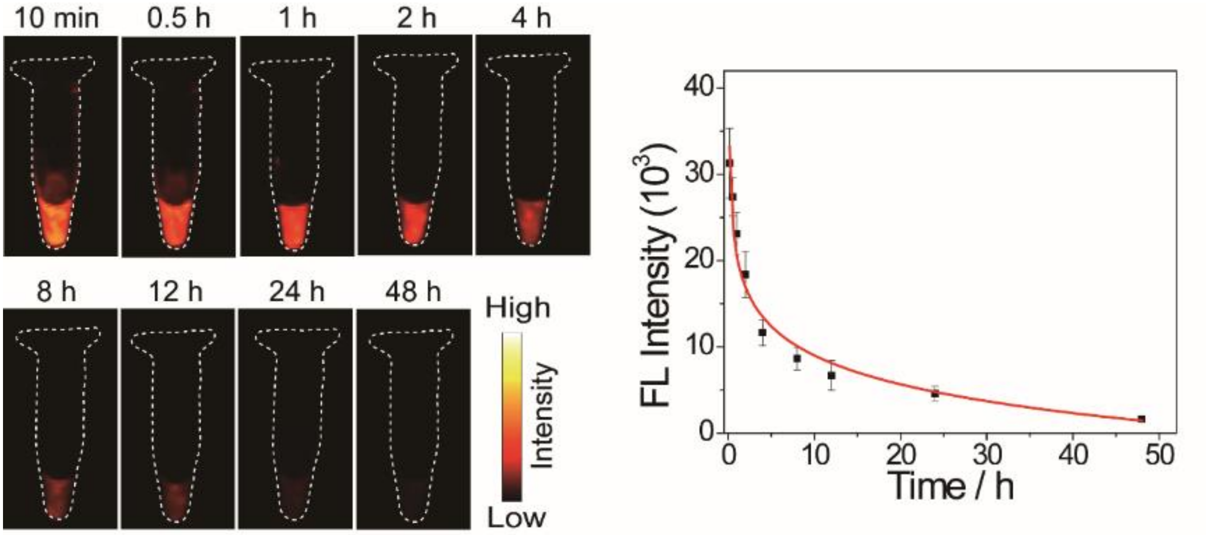

Figure S26. Fluorescence images of blood samples after IP injection of NIR-II Pdots-GnRH at different time point and the corresponding time course of fluorescence intensity during in 48 h.

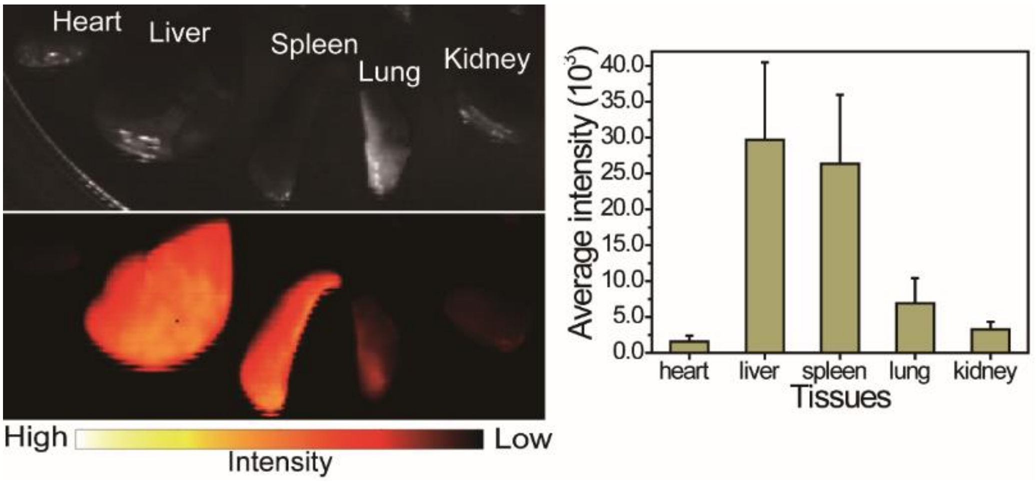

Figure S27. Biodistribution of main organs of NIR-II Pdots-GnRH-treated mice after 48 h post injection.

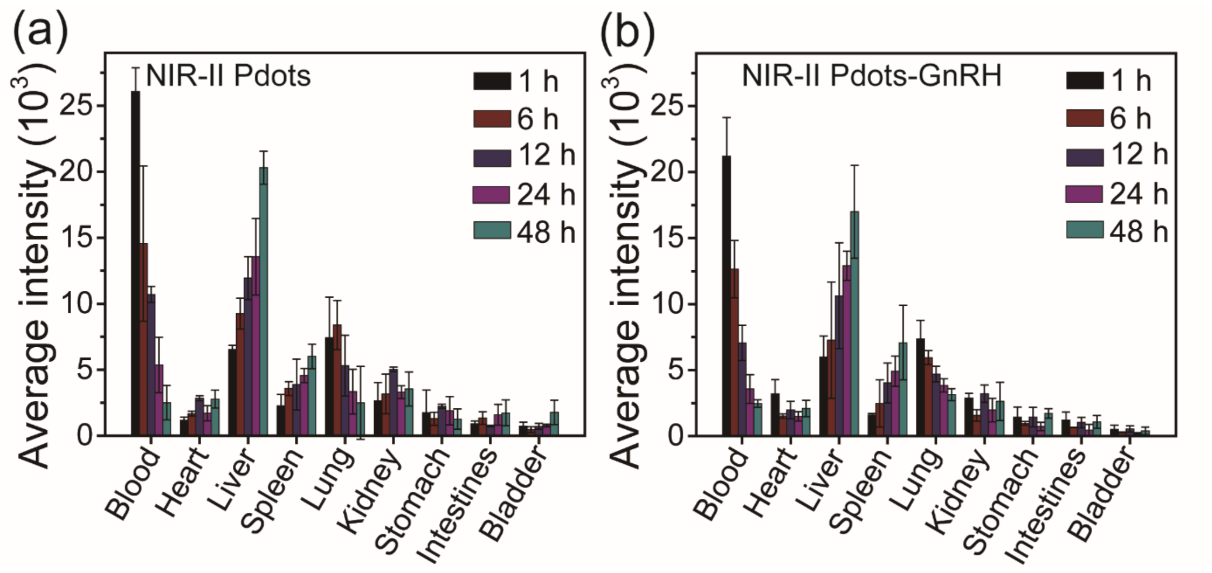

Figure S28. Biodistribution profile of NIR-II probe in mice at various time intervals (1, 6, 12, 24 or 48 h) after enous injection of (a) NIR-II Pdots and (b) NIR-II Pdots-GnRH (4 mg/kg, n = 3). The amount of probe was determined by measurement of the fluorescence intensity of tissue.

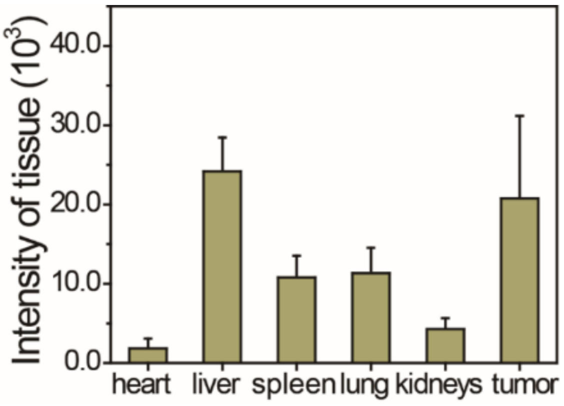

Figure S29. Quantitative results of fluorescence intensity for the ex vivo images of different organs from the subcutaneous tumor (solid tumor model)-bearing mice injected with NIR-II Pdots.

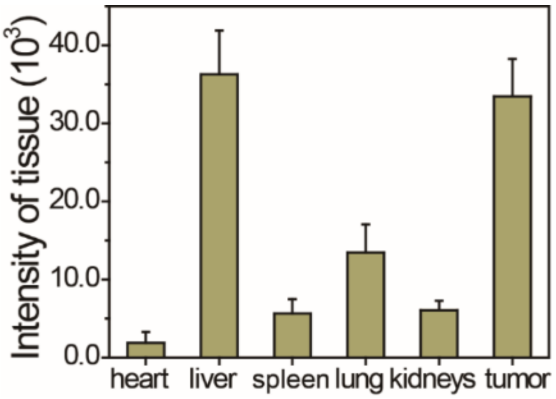

Figure S30. Quantitative results of fluorescence intensity for the ex vivo images of different organs from the tumor (solid tumor model)-bearing mice injected with NIR-II Pdots-GnRH.

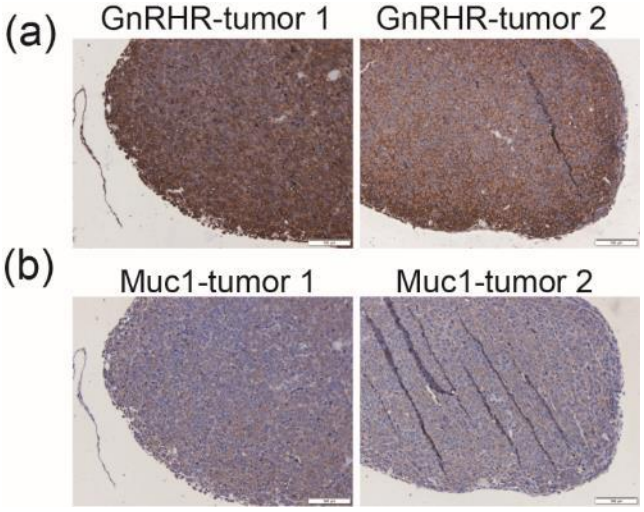

Figure S31. (a) GnRHR and (b) Muc1 expression in A2780 xenografts. Bar: 100 μm

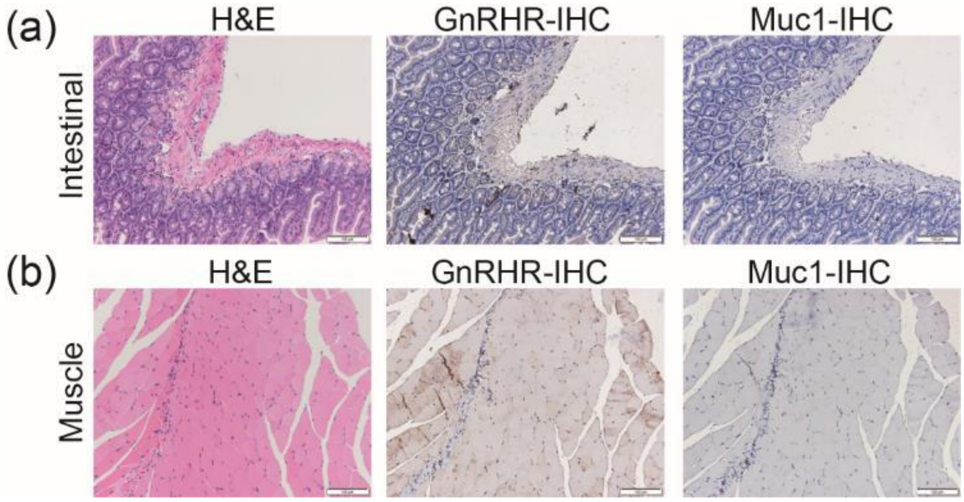

Figure S32. H&E, GnRH and Muc1 stained normal tissues, intestinal (a) and muscle (b) tissue.

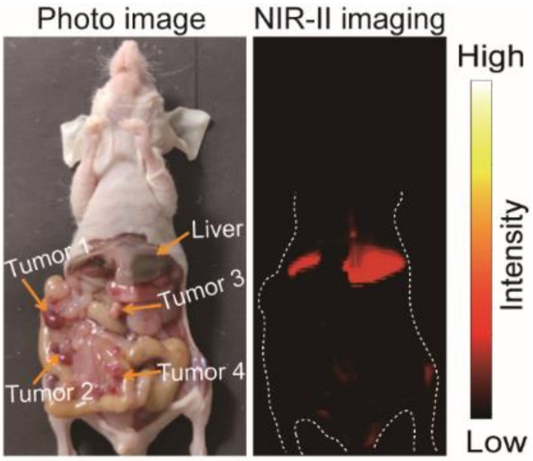

Figure S33. Optical photo of human ovarian adenocarcinoma peritoneal metastases model and the corresponding NIR-II fluorescence bioimaging results obtained at 48 h PI of NIR-II Pdots

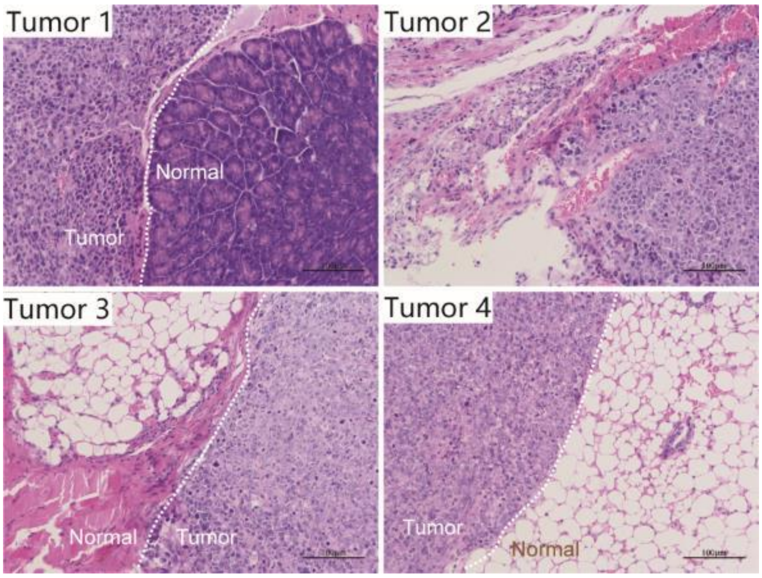

Figure S34. H&E stained images of tumor tissues shown in Figure S31.

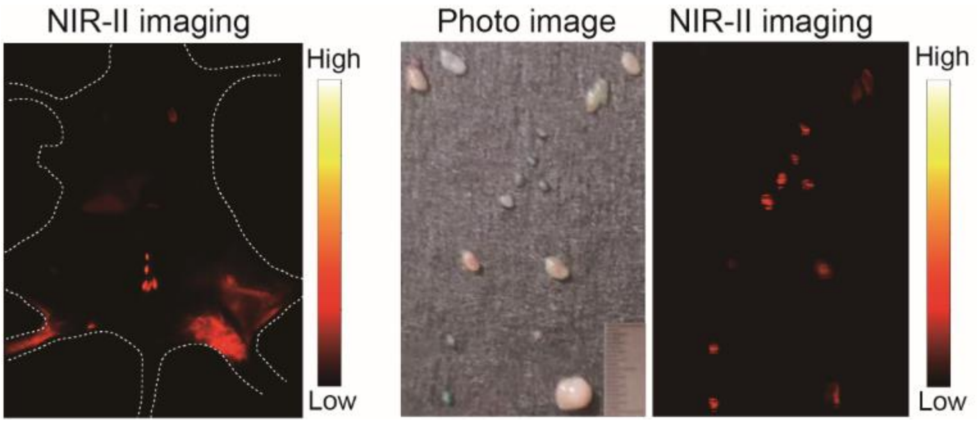

Figure S35. NIR-II fluorescence images of ovarian lymph node metastases mode obtained at 48 h PI of NIR-II Pdots-GnRH intradermal into the foot pads from both sides of the mouse (left) and the resected lymph node tissues *in vitro* (right).

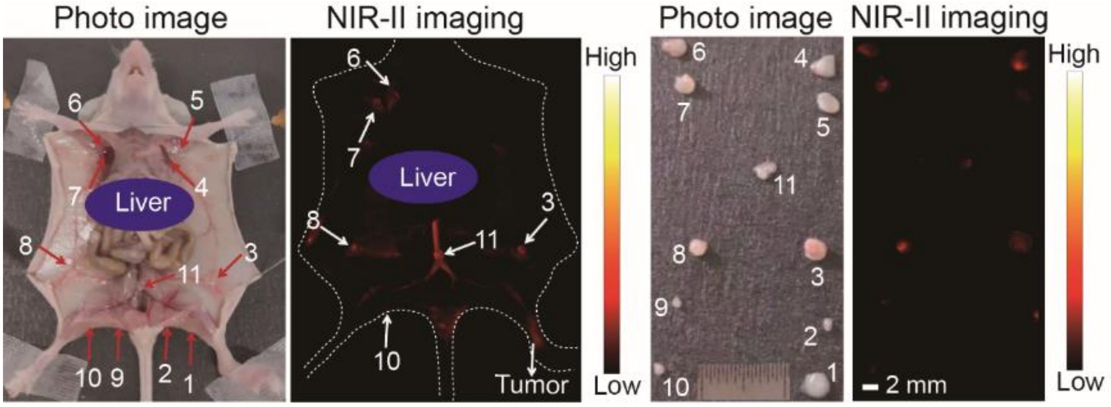

Figure S36. Optical photos and NIR-II fluorescence images of ovarian lymph node metastases mode obtained at 48 h PI of NIR-II Pdots (left) and the resected lymph node tissues (No. 1-11) *in vitro* (right).

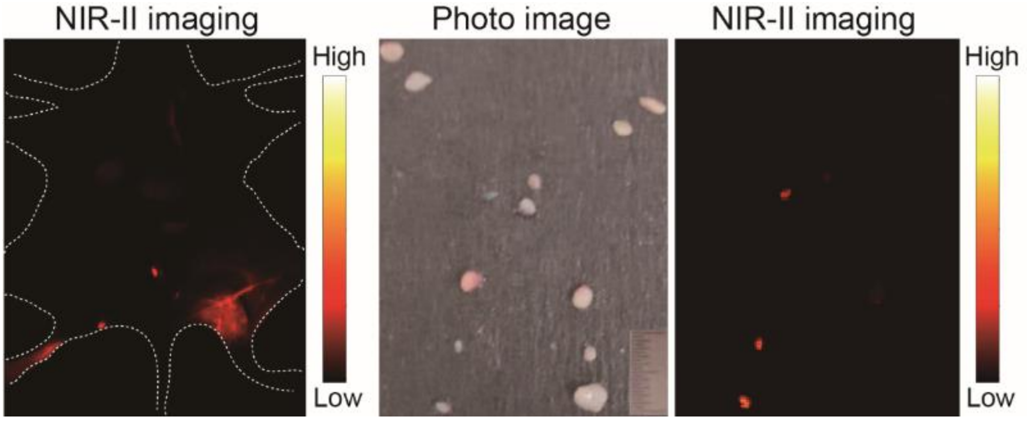

Figure S37. NIR-II fluorescence images of ovarian lymph node metastases mode obtained at 48 h PI of NIR-II Pdots intradermal into the foot pads from both sides of the mouse (left) and the resected lymph node tissues *in vitro* (right).

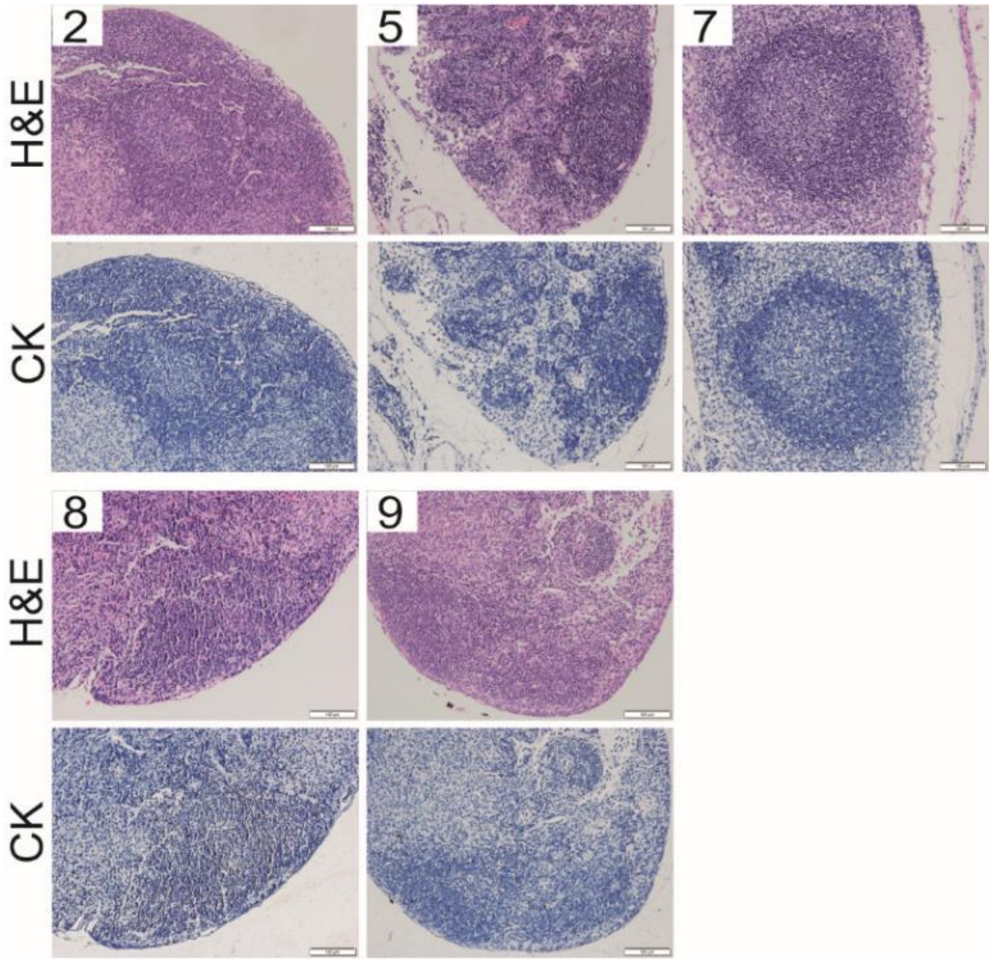

Figure S38. H&E and CK7 stained lymph node tissue (No. 2, 5, 7, 8, 9 shown in **Figure5c**) sections

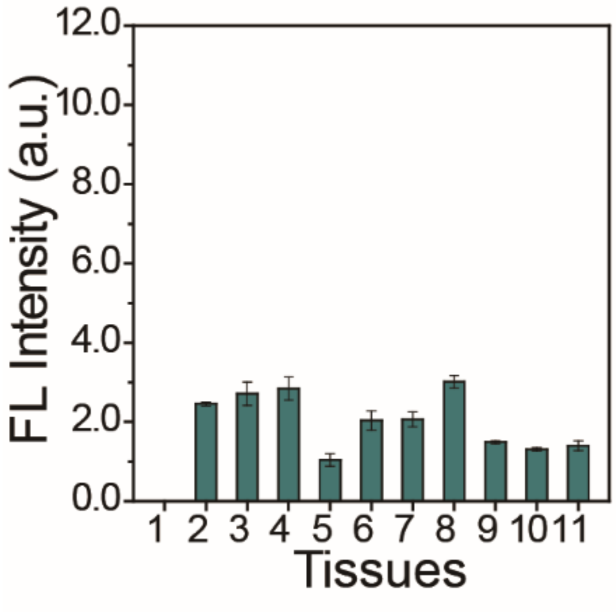

Figure S39. Average fluorescence intensity of the resected lymph node tissues shown in Figure S34.

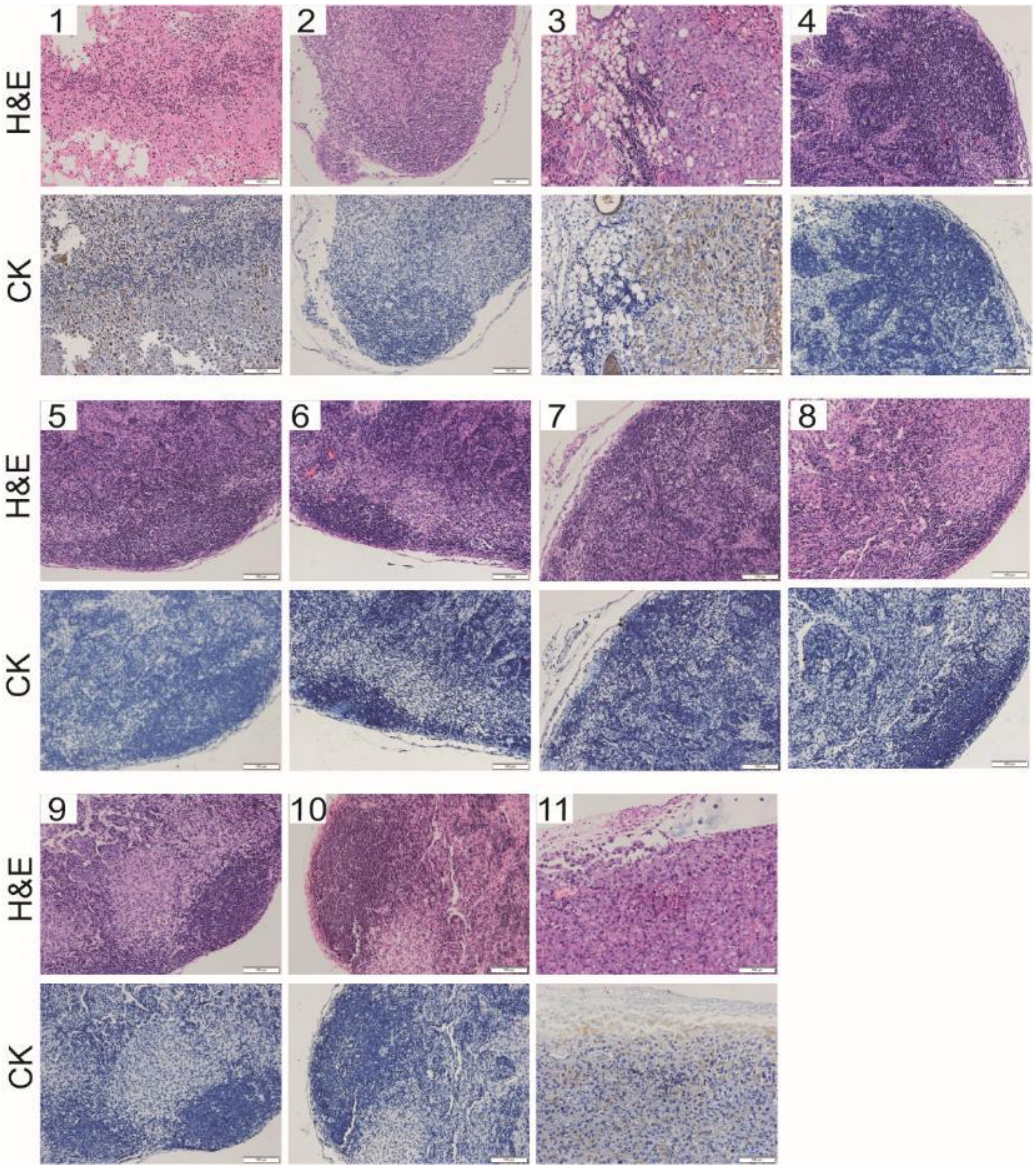

Figure S40. H&E and CK7 stained lymph node tissue (No. 1-11 shown in Figure S34) sections.

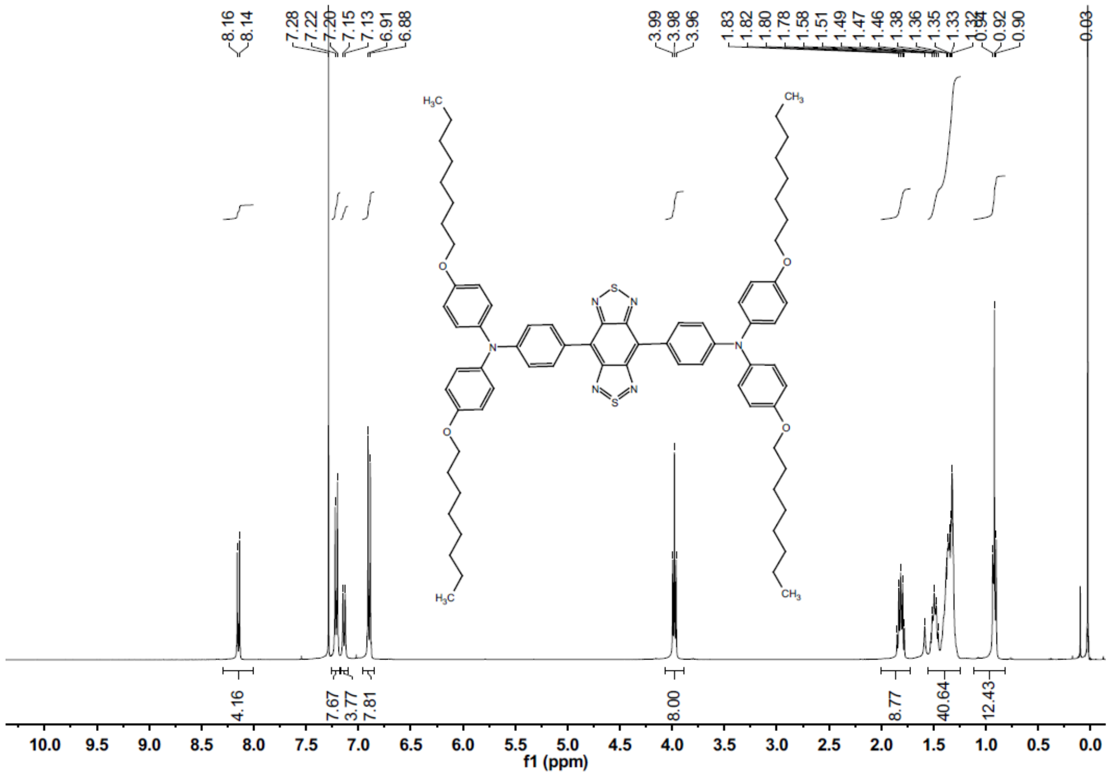

^{1}H NMR of BBTD

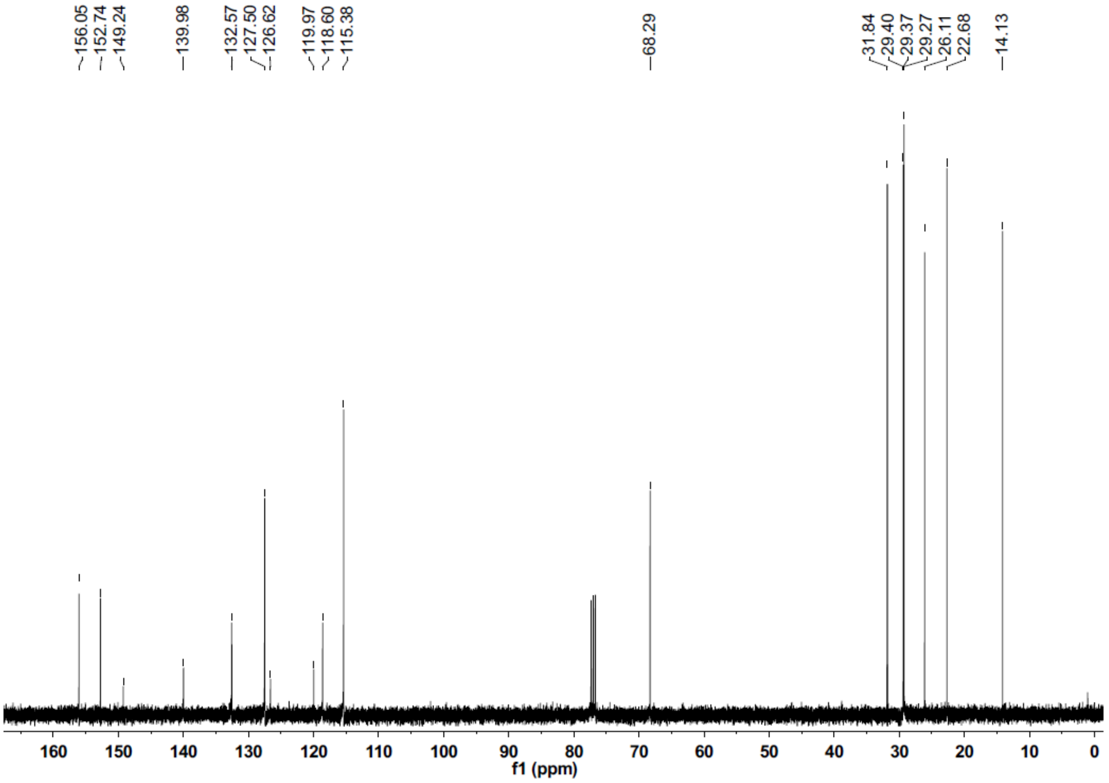

^{13}C NMR of BBTD

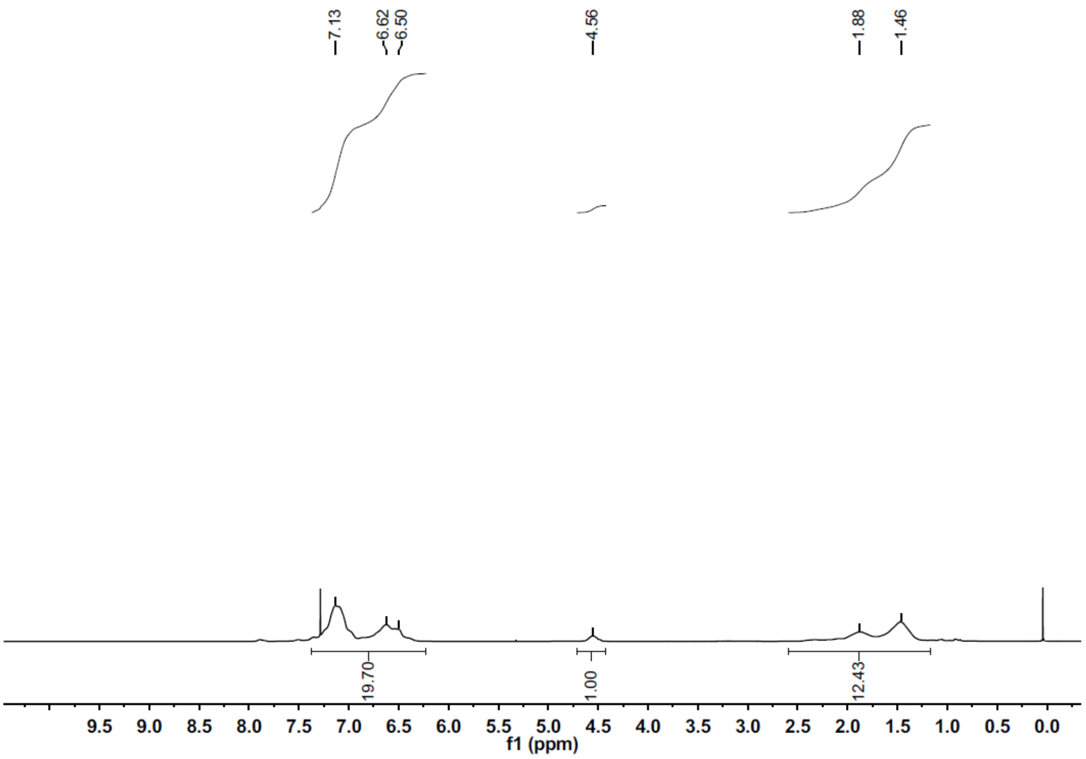

H NMR of PS-Cl

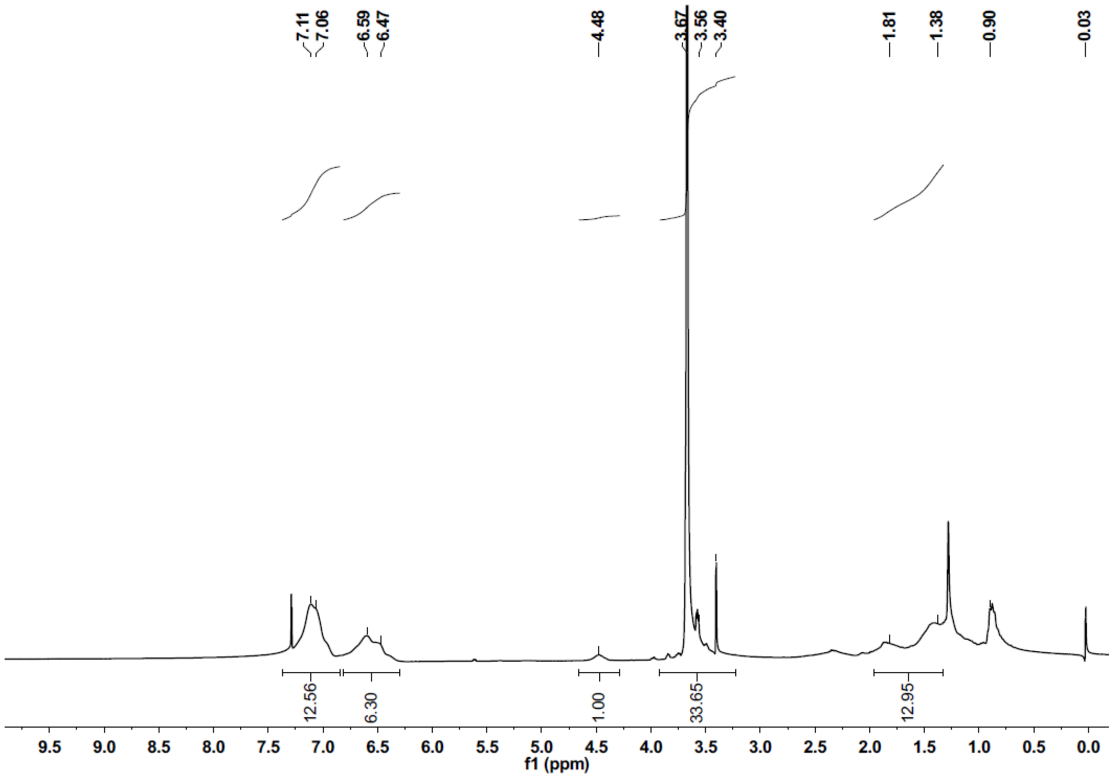

H NMR of PS-PEG2000

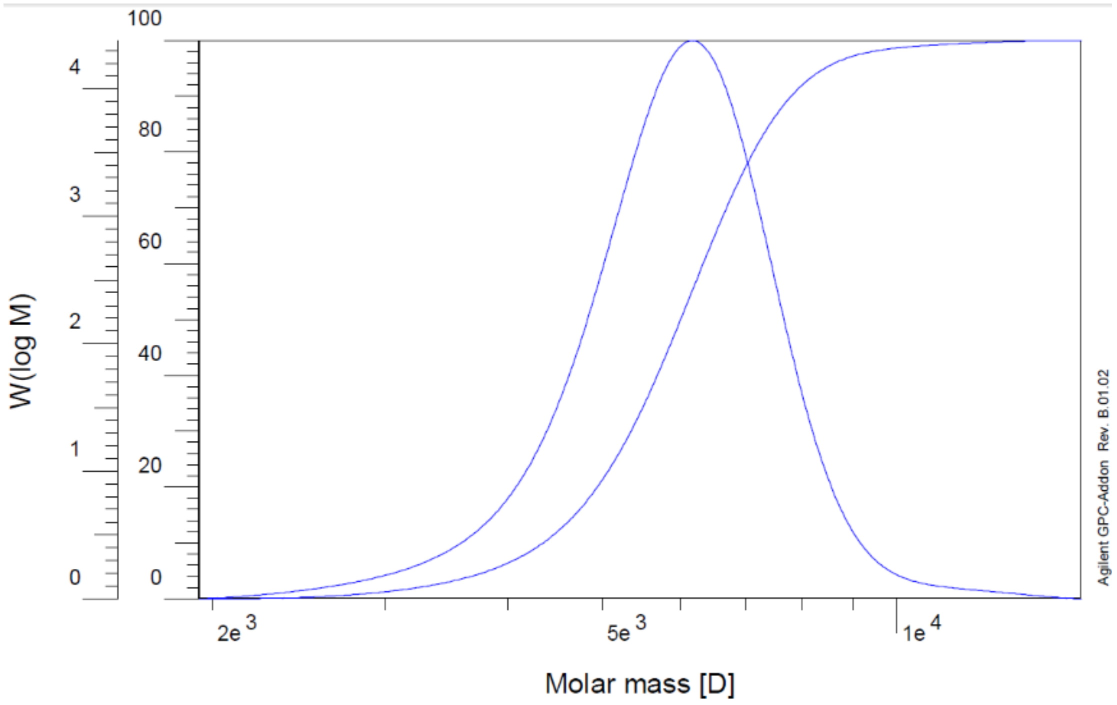

GPC of PS-Cl

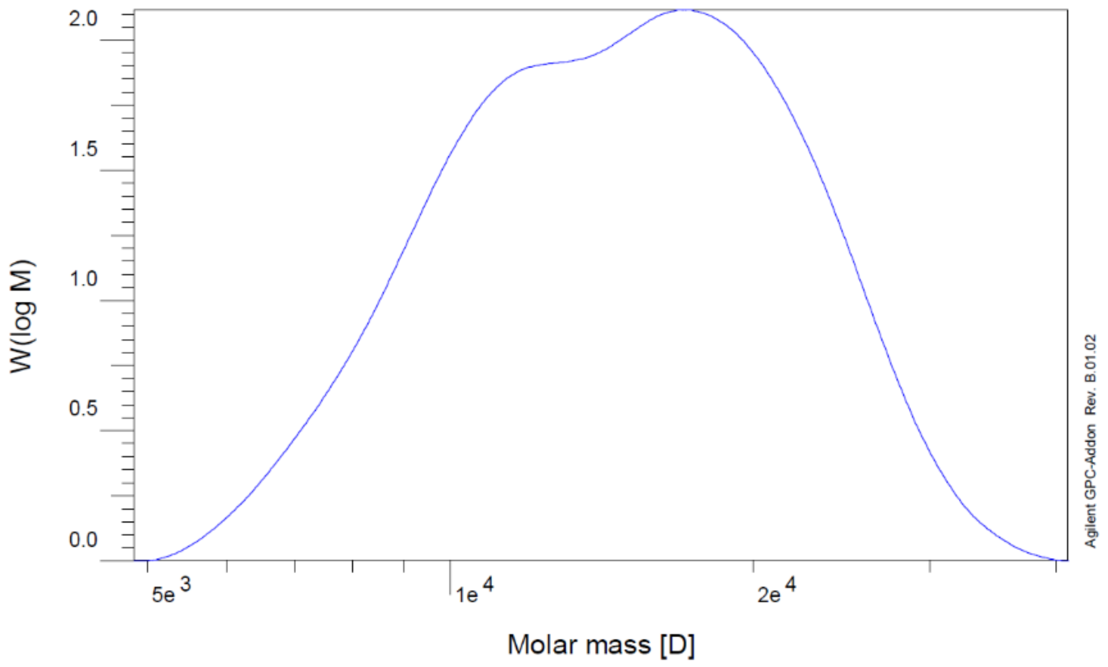

GPC of PS-PEG2000

Reference

[1] A. L. Antaris, H. Chen, K. Cheng, Y. Sun, G. Hong, C. Qu, S. Diao, Z. Deng, X. Hu, B. Zhang, X. Zhang, O. K. Yaghi, Z. R. Alamparambil, X. Hong, Z. Cheng, H. Dai, *Nat. Mater*. **2016**, *15*, 235.

[2] A. L. Antaris, H. Chen, S. Diao, Z. Ma, Z. Zhang, S. Zhu, J. Wang, A. X. Lozano, Q. Fan, L. Chew, M. Zhu, K. Cheng, X. Hong, H. Dai, Z. Cheng, *Nat. Commun.* **2017**, *8*, 15269.

[3] Q. Yang, Z. Ma, H. Wang, B. Zhou, S. Zhu, Y. Zhong, J. Wang, H. Wan, A. Antaris, R. Ma, X. Zhang, J. Yang, X. Zhang, H. Sun, W. Liu, Y. Liang, H. Dai, *Adv. Mater.* **2017**, *29*.

[4] X. D. Zhang, H. Wang, A. L. Antaris, L. Li, S. Diao, R. Ma, A. Nguyen, G. Hong, Z. Ma, J. Wang, S. Zhu, J. M. Castellano, T. Wyss-Coray, Y. Liang, J. Luo, H. Dai, *Adv. Mater.* **2016**, *28*, 6872.

[5] Q. Yang, Z. Hu, S. Zhu, R. Ma, H. Ma, Z. Ma, H. Wan, T. Zhu, Z. Jiang, W. Liu, L. Jiao, H. Sun, Y. Liang, H. Dai, *J. Am. Chem. Soc.* **2018**, *140*, 1715.

[6] B. Li, L. Lu, M. Zhao, Z. Lei, F. Zhang, *Angew. Chem. Int. Ed.* **2018**, *57*, 7483.

[7] Z. Tao, G. Hong, C. Shinji, C. Chen, S. Diao, A. L. Antaris, B. Zhang, Y. Zou, H. Dai, *Angew. Chem. Int. Ed.* **2013**, *52*, 13002.

[8] H. Wan, J. Yue, S. Zhu, T. Uno, X. Zhang, Q. Yang, K. Yu, G. Hong, J. Wang, L. Li, Z. Ma, H. Gao, Y. Zhong, J. Su, A. L. Antaris, Y. Xia, J. Luo, Y. Liang, H. Dai, *Nat. Commun.* **2018**, *9*, 1171.

[9] K. Cheng, H. Chen, C. H. Jenkins, G. Zhang, W. Zhao, Z. Zhang, F. Han, J. Fung, M. Yang, Y. Jiang, L. Xing, Z. Cheng, *ACS Nano* **2017**, *11*, 12276.

[10] Z. Sheng, B. Guo, D. Hu, S. Xu, W. Wu, W. H. Liew, K. Yao, J. Jiang, C. Liu, H. Zheng, B. Liu, *Adv. Mater.* **2018**, e1800766.

[11] S. Gao, G. Wei, S. Zhang, B. Zheng, J. Xu, G. Chen, M. Li, S. Song, W. Fu, Z. Xiao, W. Lu, *Nat. Commun.* **2019**, *10*, 2206.

[12] N. Alifu, A. Zebibula, J. Qi, H. Zhang, C. Sun, X. Yu, D. Xue, J. W. Y. Lam, G. Li, J. Qian, B. Z. Tang, *ACS Nano* **2018**, *12*, 11282.

[13] J. Qi, C. Sun, A. Zebibula, H. Zhang, R. T. K. Kwok, X. Zhao, W. Xi, J. W. Y. Lam, J. Qian, B. Z. Tang, *Adv. Mater.* **2018**, *30*, e1706856.

[14] W. Wu, Y. Yang, Y. Yang, Y. Yang, K. Zhang, L. Guo, H. Ge, X. Chen, J. Liu, H. Feng, *Small* **2019**, *15*, e1805549.

[15] G. Hong, Y. Zou, A. L. Antaris, S. Diao, D. Wu, K. Cheng, X. Zhang, C. Chen, B. Liu, Y. He, J. Z. Wu, J. Yuan, B. Zhang, Z. Tao, C. Fukunaga, H. Dai, *Nat. Commun.* **2014**, *5*, 4206.

[16] Z. Zhang, X. Fang, Z. Liu, H. Liu, D. Chen, S. He, J. Zheng, B. Yang, W. Qin, X. Zhang, C. Wu, *Angew. Chem. Int. Ed.* **2019**. DOI:10.1002/ange.201914397

[17] G. Hong, J. T. Robinson, Y. Zhang, S. Diao, A. L. Antaris, Q. Wang, H. Dai, *Angew. Chem. Int. Ed.* **2012**, *51*, 9818.

[18] O. T. Bruns, T. S. Bischof, D. K. Harris, D. Franke, Y. Shi, L. Riedemann, A. Bartelt, F. B. Jaworski, J. A. Carr, C. J. Rowlands, M. W. B. Wilson, O. Chen, H. Wei, G. W. Hwang, D. M. Montana, I. Coropceanu, O. B. Achorn, J. Kloepper, J. Heeren, P. T. C. So, D. Fukumura, K. F. Jensen, R. K. Jain, M. G. Bawendi, *Nat. Biomed. Eng.* **2017**, *1*. 1.

[19] M. Zhang, J. Yue, R. Cui, Z. Ma, H. Wan, F. Wang, S. Zhu, Y. Zhou, Y. Kuang, Y. Zhong, D. W. Pang, H. Dai, *Proc. Natl. Acad. Sci. U S A* **2018**, *115*, 6590.

[20] Y. Zhong, Z. Ma, S. Zhu, J. Yue, M. Zhang, A. L. Antaris, J. Yuan, R. Cui, H. Wan, Y. Zhou, W. Wang, N. F. Huang, J. Luo, Z. Hu, H. Dai, *Nat. Commun.* **2017**, *8*, 737.

[21] C. Cao, M. Xue, X. Zhu, P. Yang, W. Feng, F. Li, *ACS Appl. Mater. Interfaces* **2017**, *9*, 18540.
